# Discovery of Nedd4 auto-ubiquitination inhibitors

**DOI:** 10.1038/s41598-023-42997-z

**Published:** 2023-09-25

**Authors:** Darren Yong, Stuart R. Green, Pegah Ghiabi, Vijayaratnam Santhakumar, Masoud Vedadi

**Affiliations:** 1 Structural Genomics Consortium, University of Toronto, Toronto, ON M5G 1L7 Canada; 2 Department of Pharmacology and Toxicology, University of Toronto, Toronto, ON M5S 1A8 Canada; 3 Drug Discovery Program, Ontario Institute for Cancer Research, Toronto, ON M5G 0A3 Canada

**Keywords:** Ligases, Biochemistry, Ubiquitylated proteins

## Abstract

E3 ubiquitin ligases are critical to the protein degradation pathway by catalyzing the final step in protein ubiquitination by mediating ubiquitin transfer from E2 enzymes to target proteins. Nedd4 is a HECT domain-containing E3 ubiquitin ligase with a wide range of protein targets, the dysregulation of which has been implicated in myriad pathologies, including cancer and Parkinson's disease. Towards the discovery of compounds disrupting the auto-ubiquitination activity of Nedd4, we developed and optimized a TR-FRET assay for high-throughput screening. Through selective screening of a library of potentially covalent compounds, compounds **25** and **81** demonstrated apparent IC_50_ values of 52 µM and 31 µM, respectively. Tandem mass spectrometry (MS/MS) analysis confirmed that **25** and **81** were covalently bound to Nedd4 cysteine residues (Cys182 and Cys867). In addition, **81** also adducted to Cys627. Auto-ubiquitination assays of Nedd4 mutants featuring alanine substitutions for each of these cysteines suggested that the mode of inhibition of these compounds occurs through blocking the catalytic Cys867. The discovery of these inhibitors could enable the development of therapeutics for various diseases caused by Nedd4 E3 ligase dysregulation.

## Introduction

**Figure 1:**
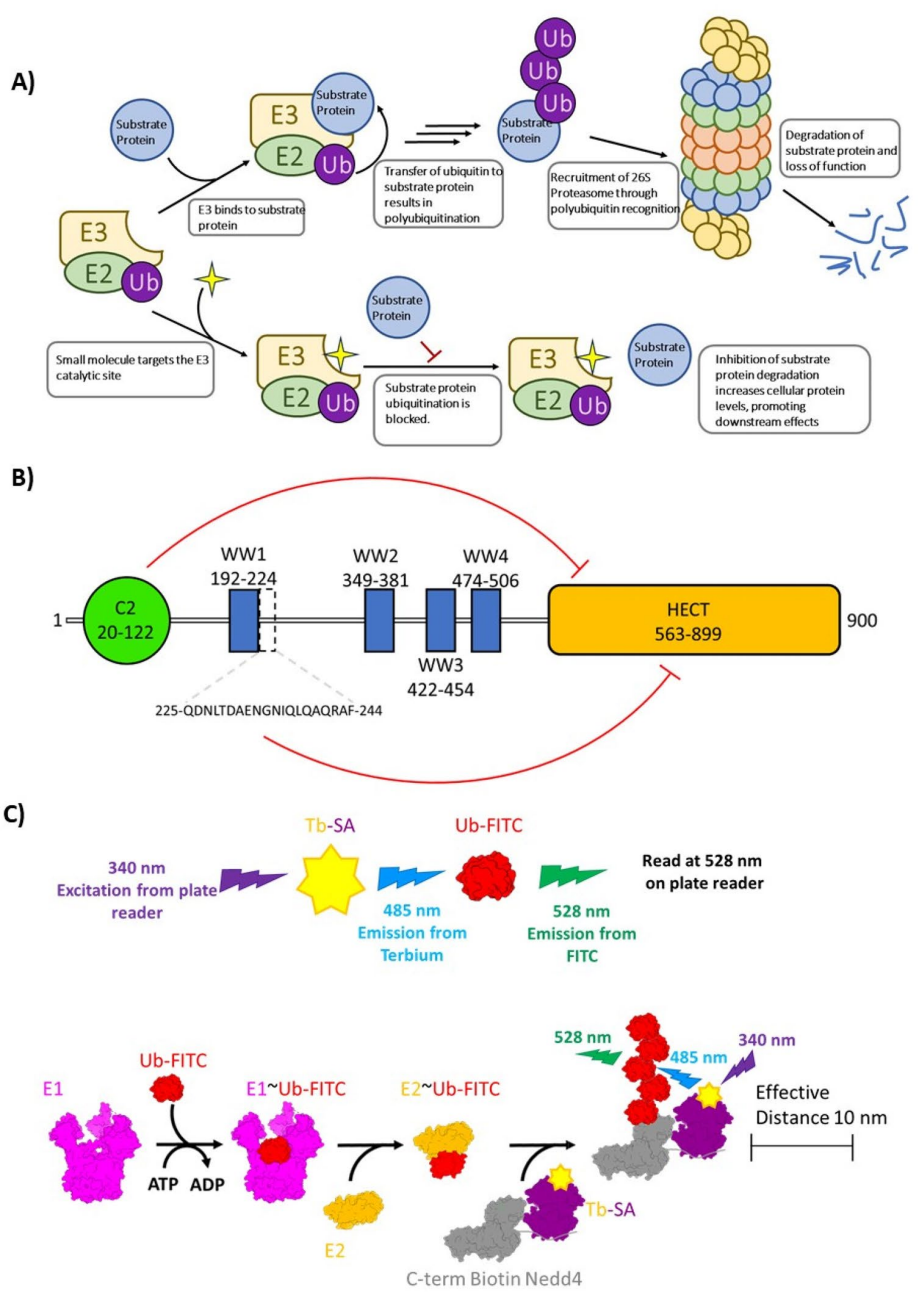
Schematics of Nedd4 function, domain structure and a TR-FRET assay for monitoring auto-ubiquitination. (**A**) The role of Nedd4 E3 ligase in degradation of target proteins and modulation of its activity by small molecule inhibitors is depicted. (**B**) Nedd4 has 3 types of folded domains. The C2 domain (20–122) drawn as a green circle, the WW domains; WW1 (192–224), WW2 (349–381), WW3 (422–454) and WW4 (474–506) drawn as blue boxes and the HECT domain (563–899) drawn as orange rectangle. The positions of the domains were predicted using the Simple Modular Architecture Research Tool (SMART) online resource by querying the full-length (1–900) Nedd4 isoform 1 sequence (NCBI accession: NP_006145.2). The sequence of the inhibitory linker peptide (225–244) between WW1 and WW2 is shown below. The red lines indicate intramolecular protein interactions with the HECT domain that inhibit E3 ligase activity. (**C**) When the terbium (Tb) labelled SA becomes excited by light at 340 nm (represented by purple in the diagram), Tb emits light at 480 nm (blue). The 480 nm emission serves as the excitation source for the FITC labelled Ub and emits light at 528 nm (green). Upon E3 ubiquitin conjugation, the terbium and FITC are brought in close proximity (within 10 nm) to allow for FRET pairing.

Ubiquitination is a post-translational modification that is catalyzed by a series of enzymes that activate, conjugate and covalently ligate ubiquitin (Ub) onto proteins^1,2^. This process is conserved across all eukaryotes and serves as a mechanism to regulate protein conformation, molecular interactions and enzymatic activity^3^ (Fig. 1A). These protein alterations in turn influence numerous fundamental cellular processes such as protein degradation, localization, transcriptional regulation, cell cycle control and other signaling pathways^4^. Despite growing understanding of the many roles performed by Ub in affecting changes in protein structure and function, ubiquitination is best known for its role in tagging proteins for proteasomal degradation. Hence, dysregulation of Ub ligases, particularly E3 enzymes, has been implicated in a wide range of diseases. The relevance of Ub E3 ligase dysregulation in disease onset has contributed to increased interest in the development of inhibitors of these enzymes from a clinical standpoint^5–7^.

Nedd4 (Nedd4-1) is the eponymous member of the Nedd4 family of HECT domain E3 Ub ligases that comprises nine related enzymes in humans^8^. Nedd4 is a 900 amino acid long protein containing several folded domains (Fig. 1B). At the N-terminus is the C2 domain that binds Ca^2+^ to induce translocation of Nedd4 to the plasma membrane through phospholipid binding^9^. The C2 domain forms intramolecular interactions with the catalytic HECT domain and serves a regulatory function by inhibiting E3 ligase activity^10^. The WW domains, named for their two conserved tryptophan residues, constitute a second type of folded domain found in Nedd4. There are 4 tandem WW domains each separated by linker regions that constitute the chief structural components responsible for protein–protein interactions between Nedd4 and substrate proteins via recognition of targets containing a PPxY motif^11,12^. These domains mediate substrate specificity and have pharmacological relevance in drug excretion through the role of the WW2 and WW3 domains in recognizing human organic anion transporter 1 for Ub ligation and subsequent degradation in the kidney^13^. The linker peptide between the WW domains provides an additional layer of catalytic regulation through its ability to bind to the HECT domain and inhibit auto-ubiquitination and is a common regulatory mechanism among the Nedd4 family of HECT E3 ligases^10^. For example, the linker between WW2 and WW3 from the Nedd4 family member WWP2 interacts with its own HECT domain and locks the N-terminus to a hinge region on the C-lobe of the HECT domain, thereby inhibiting auto-ubiquitination^14^. The conserved HECT domain at the C-terminus of the protein is present among all Nedd4 family members and functions as the catalytic module for Ub ligation to substrate proteins. The HECT E3 ligases use a conserved cysteine located on the C-lobe that catalyzes a transthioesterification reaction transferring Ub from the E2 to the HECT domain before the final transfer to the protein substrate^15^.

Owing to the variety of targets that Nedd4 can recognize, its dysregulation has been associated with several different disease states in a tissue-specific manner. Notably, Nedd4 has been implicated as both an oncogene and a tumor suppressor in different biological contexts. For example, increased levels of Nedd4 significantly downregulates PTEN and promote prostate cancer progression^16^. On the other hand, Nedd4 targets N-Myc, a proto-oncogene highly expressed in fetal brain tissues, for degradation. Otherwise, high expression of N-Myc drives the development of different cancers in the nervous, blood, and endocrine systems^17–19^. Dysregulation of Nedd4 activity has also been linked to disease pathogenesis outside of cancer. For example, ubiquitination of α-synuclein by Nedd4 promotes alpha-synuclein degradation by the endosomal-lysosomal pathway. Such reduction of α-synuclein could help protect against the pathogenesis of Parkinson disease and other α-synucleinopathies.^20^ Additionally, increased expression of a specific transcript variant of Nedd4 due to a single-nucleotide polymorphism common in certain populations has been associated with a heightened risk of the development of keloid scar formation through its downstream influence in activating the NF-κB signaling pathways^21,22^.

In general, Nedd4 and HECT domain E3 Ub ligases are attractive candidates for identifying inhibitors for pharmaceutical development due to their regulatory roles in numerous key cellular signaling pathways that are perturbed in various diseases.

Several inhibitors of the related HECT E3 ligase ITCH have been reported and have demonstrated promising therapeutic potential through their ability to reduce cancer cell-line growth and proliferation^23,24^. Indole-3-carbinol (I3C) is a natural Nedd4 inhibitor found in cruciferous vegetables such as broccoli and cabbage. Computational binding studies of I3C to the HECT domain suggest binding occurs at a pocket in the N-lobe of the HECT domain away from the C-lobe catalytic site^25^. This binding site is associated with the Ub exosite of the HECT domain. This site accommodates a non-covalently bound Ub and promotes poly-Ub chain elongation during auto-ubiquitination^15,26^. Structural analogs of I3C have demonstrated IC_50_ values as low as 2 µM^25^. Another study has demonstrated that indole-based fragments derivatized for electrophilic covalent adduction preferentially ligate to the non-catalytic Cys627 residue on the N-lobe over Cys867 residue and cause Nedd4 inhibition by interfering with Ub binding in the exosite binding pocket^27^. The development of covalently binding derivates of I3C inhibitor in this site may enhance pharmaceutical development over non-covalent binders by enabling irreversible inhibition of Nedd4 ubiquitination kinetics^28^.

Here, we report the development of an optimized TR-FRET assay for high-throughput screening of Nedd4 and discovery of two covalent Nedd4 inhibitors targeting critical catalytic residue, Cys867. Our discovery could significantly contribute to the discovery of more potent and selective Nedd4 ligands towards development of therapeutics for various cancers and other diseases.

## Materials and methods

### Reagents

Terbium labeled streptavidin (SA) (cat no. PV3965) and fluorescein isothiocyanate (FITC) labeled ubiquitin (cat no. PV4378) were purchased from Thermo Fisher Scientific (Waltham, MA, USA) (Supplementary Table 1). A subset of a covalent library of 3200 small fragments (Enamine, Kyiv, Ukraine) containing an electrophilic alpha-chloroketone warhead was selected to screen Nedd4.

### Protein expression and purification

N-terminal His-tagged Uba1 (residues 1–1058) was expressed in *Spodoptera frugiperda* 9 (*Sf9*) cells following recombinant baculovirus transfection, while constructs for N-terminal His-tagged UbcH5a (residues 1–148) and N-terminal His-tagged Nedd4 with a C-terminal biotin acceptor peptide were expressed in *E. coli BL21 (DE3)*. Biotinylated Nedd4 proteins were expressed in *E. coli BL21 (D3)-BirA* cells, allowing for biotinylation of Nedd4 during protein expression through addition of 10 µg/mL biotin. N-terminally His-tagged WWP1 (residues 349- 922) was expressed in *E. coli* BL21 (DE3) and WWP2 (residues M 1-E 870, isoform 1) was expressed in *Sf9* with an N-terminal Avi-tag for in-cell biotinylation and C-terminal His-tag. All His-tagged proteins were purified by gravity-flow Ni^2+^-nitrilotriacetic acid (NTA) affinity chromatography (Cat. No. Qiagen 30210) followed by either gel filtration and/or ion-exchange chromatography. The His-tags for Uba1 and UbcH5a were cleaved by thrombin and TEV proteases, respectively, and removed by a second round of Ni^2+^-NTA affinity chromatography. Following Ni^2+^-NTA chromatography, His-tagged Nedd4 proteins were purified by gel filtration and anion exchange. Complete descriptions of the protocols used in purifying and processing the proteins are provided in supplementary materials and “Materials and methods” section.

### TR-FRET auto-ubiquitination assay

All time-resolved fluorescence resonance energy transfer (TR-FRET) auto-ubiquitination assays were performed in a final volume of 10 µL in PerkinElmer 384-well flat bottom white OptiPlate™ polystyrene microplates (PerkinElmer Cat. No. 6007290). Optimized reaction conditions were as follows: 50 nM of Uba1, 125 nM of UbcH5a, 130 nM of N-terminal His-tagged-Nedd4 (residues 1–900) C-terminal biotinylated, 300 nM of Ub-FITC and 50 nM of Tb-SA in assay buffer (150 mM NaCl, 20 mM HEPES pH 7.5, 0.00063% NP-40, 0.1 mM TCEP, 2 mM ATP-MgCl_2_) (Supplementary Table 2). All assay buffer component concentrations were selected by optimizing for TR-FRET signal across serial dilutions for the individual components (Supplementary Fig. 1). A low concentration of NP-40 detergent (0.00063% v/v) was chosen for the Nedd4 auto-ubiquitination assay since this concentration yielded optimal terbium emission at 485 nm after 340 nm excitation (Supplementary Fig. 2). Concentrations of Ub activating (E1) and conjugating (E2) enzymes and fluorophores were selected to provide sufficient TR-FRET signal intensity for compound screening without excessive reagent consumption (Supplementary Fig. 3). Reaction mixes containing all assay components except ATP-MgCl_2_ were preincubated with either library compounds dissolved in 1% DMSO or DMSO alone at RT for up to 2 h. To initiate the auto-ubiquitination reaction, 2 mM ATP-MgCl_2_ was added and incubated at 37 °C for 60–90 min. TR-FRET signal was measured in a Synergy 4 microplate reader (BioTek) using a 340/30 nm excitation filter and measuring the emission ratio using 485/20 nm and 528/20 nm emission filters. Fluorescence measurement was performed over a 400 µs window following a 100 µs delay post-excitation (Fig. 1). Similarly, TR-FRET assays were used to measure the auto-ubiquitination activity of two other Nedd4 family HECT E3 ligases (WWP1 and WWP2) using alternative optimized buffer and fluorophore concentrations to test Nedd4 inhibitor specificity (Supplementary Table 2).

All experiments were performed in triplicate (n = 3), and plotted values represent the average of three replicates with the standard deviation. TR-FRET signal refers to background values subtracted using control wells containing Tb-SA and Ub-FITC. Data were plotted using GraphPad 9.1.2 (GraphPad, La Jolla, CA) and fitted to a four-parameter sigmoidal dose–response curve. Where applicable, the dose–response was normalized and expressed as a percentage of auto-ubiquitination activity relative to the DMSO control.

### Z′-factor determination

The Z′-factor was determined by preparing reactions in the presence or absence of 2 mM ATP-MgCl_2_ as described above for 88 wells each. A time course experiment demonstrated that the TR-FRET signal increased linearly for up to 1 h after initiating the reaction by adding ATP-MgCl_2_ (Supplementary Fig. 4A). The Z′-factor was determined to be 0.75, indicating that the Nedd4 TR-FRET assay is suitable for high throughput screening (Supplementary Fig. 4B).

### Western blotting for ubiquitination and biotinylated Nedd4

Western blots were performed to demonstrate the ubiquitination of Nedd4 under the standard reaction conditions described in the TR-FRET auto-ubiquitination assay section, and inhibition of auto-ubiquitination by compounds **25** and **81**. For testing the compounds, proteins were incubated with varying concentrations of **25** or **81** for 2 h at RT using 1% DMSO as a positive control. The auto-ubiquitination reactions were initiated with the addition of 2 mM ATP-MgCl_2_. Another control reaction was performed without the addition of ATP-MgCl_2_ for comparison (negative control). The reactions were allowed to proceed for 1 h at 37 °C before the reactions were terminated by the addition of 4 × SDS sample buffer to working concentrations followed immediately by 10 min heating at 100 °C to denature the proteins. Samples were loaded onto a 4–20% Tris–Glycine (Novex Cat. No. XP04205BOX) gel and run for 1 h at 150 V. Proteins were then transferred to a PVDF membrane (Bio-Rad, Cat#: 1620177) and subsequently blocked with 5% skim milk powder before overnight incubation in a cold room with a mouse anti-ubiquitin antibody (Abcam 7254) primary diluted to 1:1000 in a 5% BSA solution. After rinsing the blots, a fluorescently labeled (excitation 685 nm, using emission filter of 710–730 nm) donkey anti-mouse secondary IgG antibody (1:5000, IRDye® 680RD LI-COR #926-68072) was applied to the blot for 30 min rinsed and a fluorescently labeled streptavidin (1:5000, IRDye^®^ 800CW Streptavidin LI-COR # 926-32230) was applied to the blot for another 30 min before reading (excitation 785 nm and emission filter 812–832 nm) using a LI-COR Odyssey Clx imaging system to visualize the membrane through IR fluorescence.

### LC–MS/MS preparation

LC–MS/MS was used to confirm covalent adduction and site identification for two positive hits generated from the Nedd4 inhibition screen that demonstrated low IC_50_ values. 30 µg of N-terminal His-tagged and C-terminal biotinylated Nedd4 (residue 1–900) was mixed with 200 µM of compound **25** or **81** (Enamine Cat. No. Z65886524 and Z1205657985) solubilized in 1% DMSO and incubated at RT for 3 h. Samples were run on SDS-PAGE and stained with Coomassie Brilliant Blue R-250 (Thermo Fisher Scientific cat no. 20278). Gels were destained in water until the desired contrast was reached. The Nedd4 bands were cut and stored in 1% acetic acid at 4 °C until use. Samples were submitted to the SPARC Molecular Analysis Centre at the Hospital for Sick Children (Toronto, ON, CA) for LC–MS/MS analysis. Each gel band was cut into 3 pieces and digested with 0.8 µg pepsin, 0.6 µg trypsin and 0.6 µg chymotrypsin separately. The peptides obtained through the proteolytic digest were combined then lyophilized and resuspended in 0.1% formic acid. These samples were then subjected to MS/MS spectroscopy by an EASY-nLC 1200 nano-LC system + Thermo Scientific Orbitrap Fusion Lumos Tribrid mass spectrometer. MS data was analyzed using Scaffold PTM 4.0.1.

### Cysteine adduction site visualization

Modified cysteine residues identified through mass spectrometry were mapped to the crystal structure of the human Nedd4 HECT domain in complex with Ub at the exosite (PDB ID: 2XBB)^26^. The modified cysteines were visualized using ChimeraX 1.3^29^.

## Results

### Nedd4 TR-FRET auto-ubiquitination assay development

To screen a small molecule library and identify Nedd4 auto-ubiquitination inhibitors, a TR-FRET assay was developed to measure Nedd4 auto-ubiquitination activity. This assay quantifies ubiquitin chain elongation on Nedd4 through the increase in TR-FRET signal from close proximity of biotinylated Nedd4 bound Tb-SA and Ub-FITC upon ligation of Ub-FITC to Nedd4 (Fig. 1C). Conversely, a reduced TR-FRET signal following compound incubation is interpreted as inhibition of Nedd4 auto-ubiquitination activity.

To determine the optimal assay buffer for Nedd4 autoubiquitination activity, different buffer concentrations and components were explored to maximize the TR-FRET signal within reasonable boundaries mimicking physiological conditions. Assay buffer conditions were optimized for TR-FRET signal by varying the concentrations of NaCl, HEPES buffer (pH 7.5), DTT, TCEP, Triton X-100 and DMSO (Supplementary Fig. 1). TCEP was used as a reducing agent since its use resulted in a higher TR-FRET signal over a wider range compared to DTT. Concentrations of Triton X-100 as low as 0.02% were shown to result in a significant decrease in TR-FRET signal and hence this detergent was omitted from further testing. Instead, a small amount of NP-40 detergent was used in future assays as this was found to be essential for optimal terbium emission and reducing the surface tension at low volumes (Supplementary Fig. 2).

The reaction conditions were further optimized by varying the concentration of each enzyme and fluorophore reagent (Supplementary Fig. 3). All concentrations of Uba1 (E1) and UbcH5a (E2) tested yielded comparable TR-FRET signal indicating that their activities are not the rate limiting step in the auto-ubiquitination reaction (Supplementary Fig. 3A,B). Varying amounts of Nedd4 demonstrated increasing TR-FRET signal up to 250 nM. Further increase in Nedd4 concentration had little effect on the signal (Supplementary Fig. 3C). TR-FRET signal was most sensitive to alterations in the concentration of the two fluorophores; Ub-FITC and Tb-SA. Increasing concentrations of Ub-FITC up to 5000 nM did not result in saturation of Nedd4 auto-ubiquitination signal (Supplementary Fig. 3D). However, a concentration of only 300 nM Ub-FITC yielded sufficient signal for reproducible results and was used for future assays. Optimizing for Tb-SA concentration demonstrated an optimum TR-FRET signal at 125 nM with decrease in TR-FRET signal beyond this point (Supplementary Fig. 3E). Tb-SA at 50 nM was used in the screening of compounds since this concentration resulted in sufficient signal-to-noise while minimizing reagent consumption.

### TR-FRET Nedd4 auto-ubiquitination assay validation

**Figure 2:**
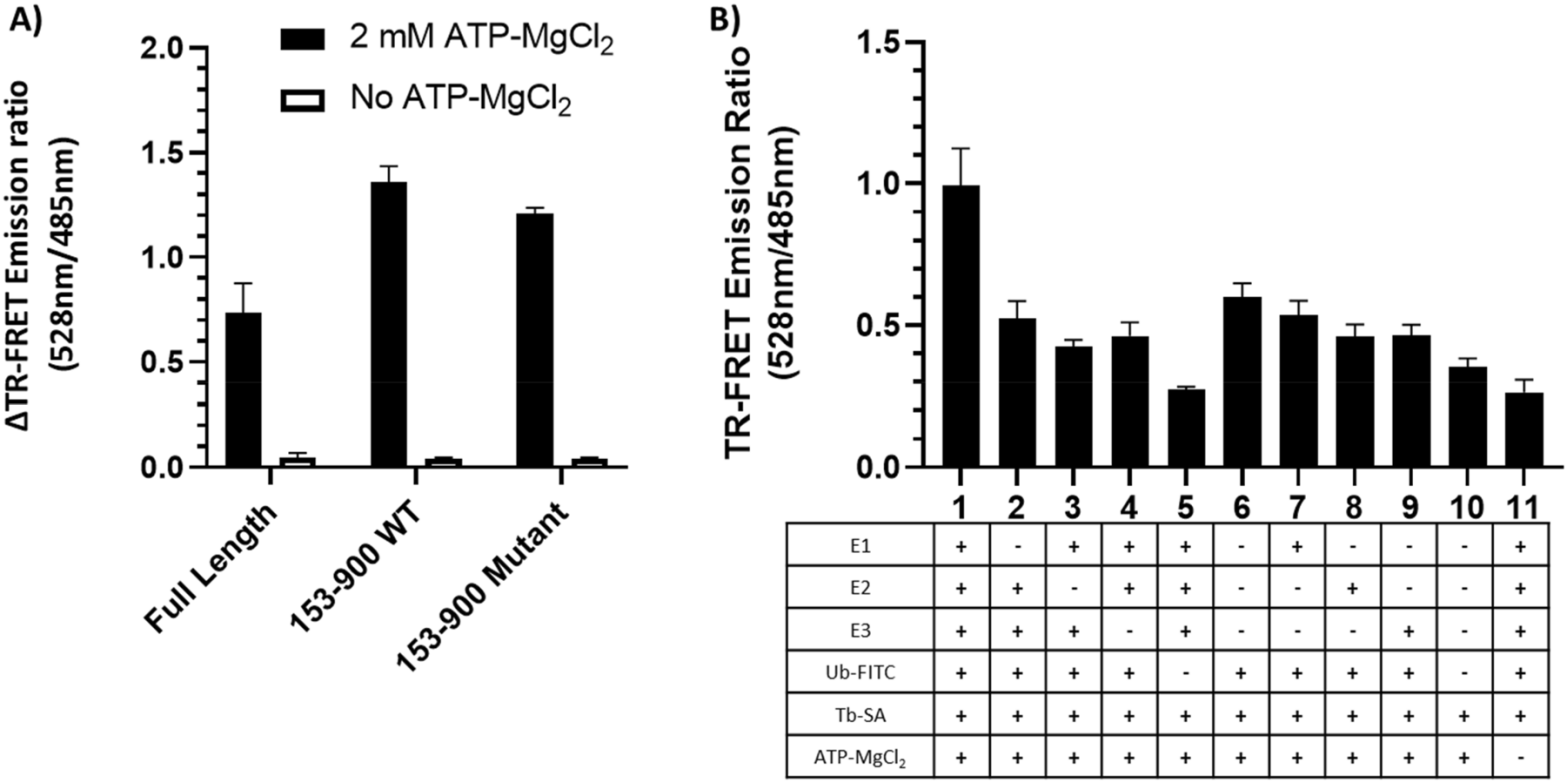
Assessment of TR-FRET signal for detecting Nedd4 auto-ubiquitination activity. (**A**) Plotting TR-FRET emission ratio above background (528/485 nm) of the full-length WT (1–900) and truncated variants (153–900) lacking the inhibitory C2 domain with and without the addition of 2 mM ATP-MgCl_2_. The mutant variant of the truncated protein also lacks the inhibitory linker peptide (225–244). (**B**) Removing reaction components demonstrates a consistent decrease in the TR-FRET 528/485 nm emission ratio with the loss of one or more reagents, demonstrating that all reaction components are essential for ubiquitination to occur during the assay. All values are from the experiments performed in triplicate, and are presented as mean ± standard deviation. The composition of each reaction (1 to 11) is presented in the corresponding column in the table below the numbers.

Validation of the TR-FRET assay as a tool for measuring Nedd4 auto-ubiquitination activity was performed by comparing the TR-FRET signal generated from full-length wild-type (WT) Nedd4 in comparison to truncated Nedd4 variants lacking regions known to have regulatory roles in auto-ubiquitination activity. Use of a truncated version of the Nedd4 protein (153–900) lacking the inhibitory N-terminal C2 domain demonstrated increased activity as expected (Fig. 2A). The stretch of peptides from 225 to 244 has been previously reported to inhibit Nedd4 ubiquitination through synergistic effects of the inhibitory C2 domain^14,16^ (Fig. 1). However, in our hand, the Nedd4 153–900 mutant with deletion between 225 and 244 did not show significant difference in activity from the wild-type Nedd4 (153–900). A drop out experiment was conducted to demonstrate that Nedd4 autoubiquitination requires all the components in the ubiquitination cascade (e.g., E1, E2, ATP-MgCl_2_). As expected, dropping out any of the components involved in the ubiquitination cascade resulted in a significant decrease in the 528/485 nm emission ratio, suggesting the TR-FRET signal can serve as an indirect measure of Nedd4 auto-ubiquitination activity (Fig. 2B). Background subtracted TR-FRET signal was calculated for all experiments by subtracting the 528/485 nm emission ratio obtained from wells containing all assay components except for the E1, E2 and Nedd4 protein. The amenability of the assay for high-throughput screening was further tested by double checking the linearity of initial velocity, confirming linear increase in activity for at least 60 min (Supplementary Fig. 4A), and running the reactions for 60 min in a 384-well format in the presence and absence of ATP indicating a Z′-Factor of 0.75 (Supplementary Fig. 4B).

### Screening for inhibitors

**Figure 3:**
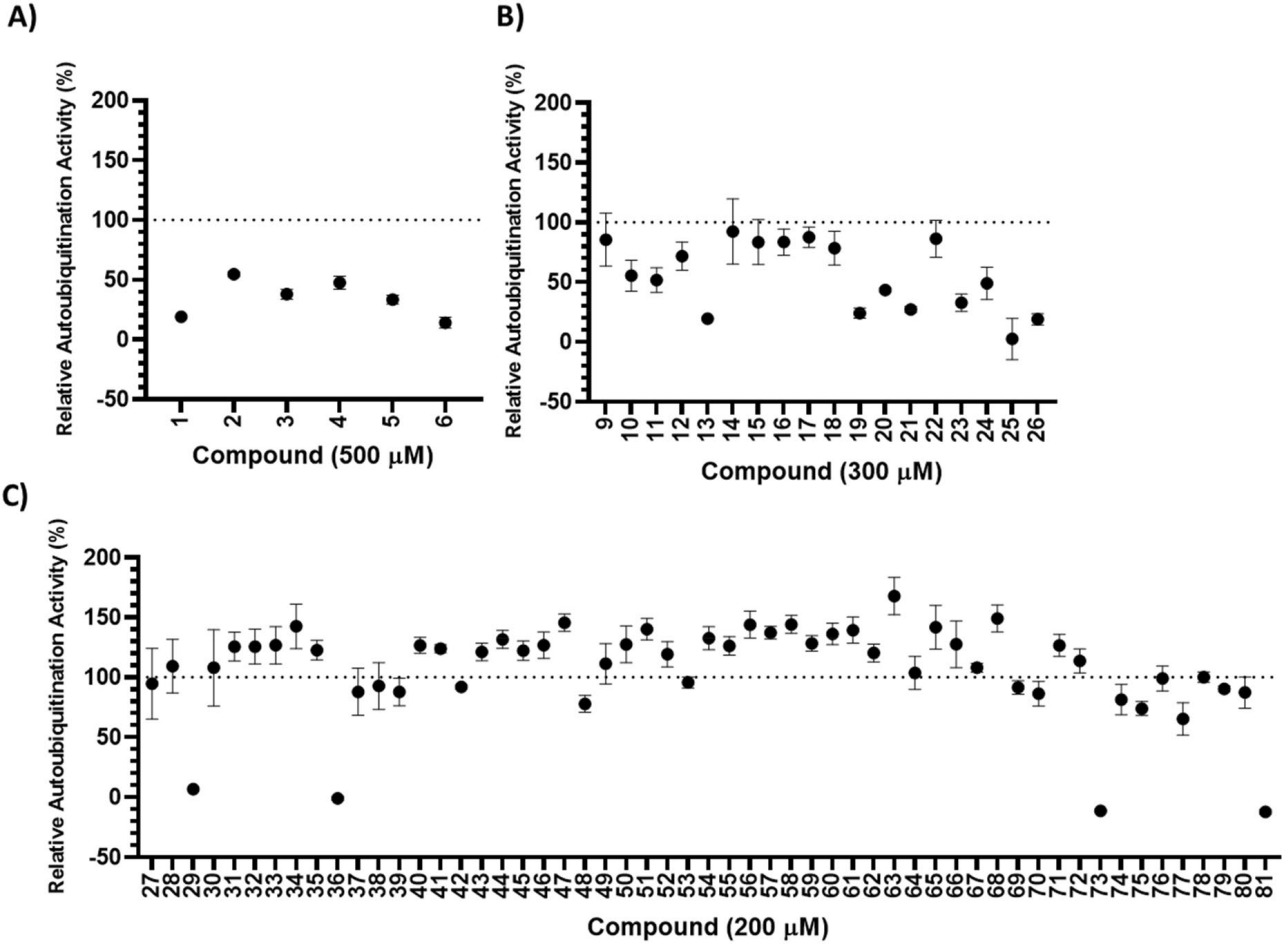
Initial screening of compounds for full-length Nedd4 auto-ubiquitination inhibition. (**A**) The first set of compounds were screened at 500 µM. (**B**) The second set of compounds were screened at 300 µM. (**C**) The final set of compounds were screened at 200 µM. Screening of compounds were performed in triplicate, and are presented as mean ± standard deviation. Information on each set of compounds is presented in supplementary Table 3 and Source Data 1. Percent activity was calculated as ratio of the background subtracted TR-FRET signal at any given concentration of a potential inhibitor and the background subtracted TR-FRET signal in the absence of compound.

**Figure 4:**
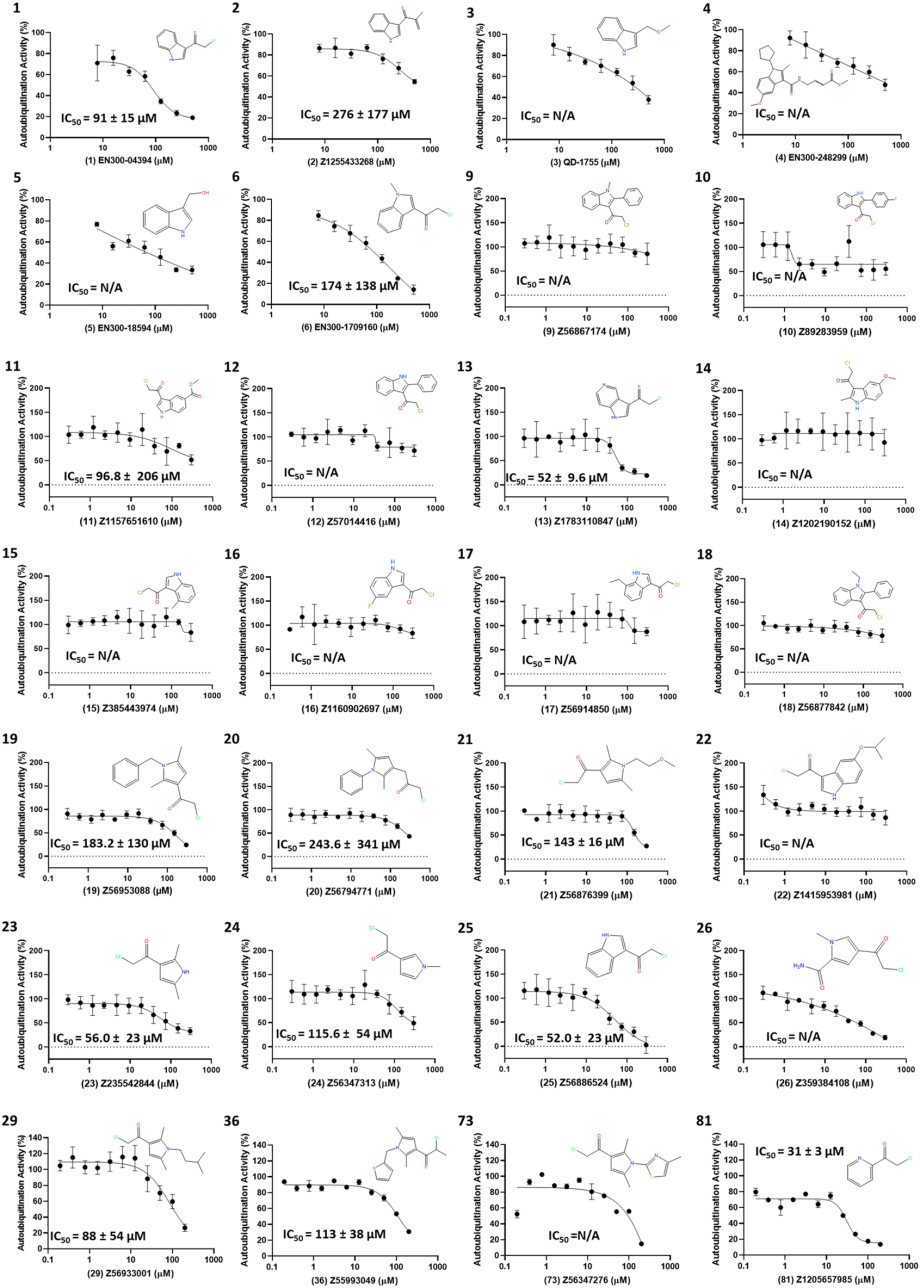
Covalent inhibitors of full-length Nedd4 auto-ubiquitination. Compounds were chosen from the initial screen when demonstrating > 50% inhibition at the single high concentration initially tested. Experiments were performed in triplicate following a 2 h RT incubation in the selected compound at varying concentrations with data presented as the relative auto-ubiquitination activity compared to the uninhibited conditions. Data were analyzed and plotted using 4-parameter non-linear regression. The relative IC_50_ values ± standard deviation are displayed on curves whose data followed a clear sigmoidal response over the tested concentration range. Catalog numbers for the compounds (and arbitrary number in parentheses) are shown below the graph and molecular structures are presented alongside the data. All compounds were obtained from Enamine with the exception of **3** (QD-1755) which was purchased from Combi-Blocks. Information on all compounds is presented in supplementary Table 3 and Source Data 1.

The Nedd4 HECT domain utilizes a catalytic cysteine for auto-ubiquitination and another non-catalytic cysteine that enhances ubiquitination through binding an allosteric Ub^27^. Therefore, potential inhibitors were selected from a subset of an in-house small fragment library of 3200 compounds containing an electrophilic alpha-chloroketone group with the ability to form adducts to cysteine residues. Using the optimized TR-FRET Nedd4 auto-ubiquitination assay, a total of 79 compounds were screened first at a single high concentration of 500 µM. This collection consisted of the HECT domain inhibitor I3C (**5**) and structural analogs thereof^25,27,30^ (Fig. 3A). Interestingly, all other compounds in this initial first set, which were screened at 500 µM, elicited at least a 40% loss of Nedd4 activity relative to the control. To overcome the challenge of the selecting the most potent compounds out of the high number of hits, we re-screened 18 compounds at lower concentration of 300 µM (Fig. 3B) and the rest (55 compounds) at even lower concentration of 200 µM (Fig. 3C) to triage and rank-order compounds. The list of all 79 compounds, chemical structures and potencies are provided in the Supplementary Table 3 (Source data 1). The data are presented at different concentrations as were confirmed. Here on, we assign an arbitrary unique identifier number to each compound (in bold) to simplify referring to each compound throughout the text. Performing the assay on a set of known and suspected inhibitors helped to demonstrate the amenability of this assay for screening Nedd4 inhibitors and provided a baseline level of inhibition. Compound **5** corresponds to I3C and caused about 67% reduction in Nedd4 activity at concentrations over 200 µM (Figs. 3A and 4). Compound **4** is another previously reported inhibitor^27^ that covalently adducts to the non-catalytic Ub binding site on the N-lobe of Nedd4, and here was observed to cause a 52% reduction in activity at the highest concentration over a 2 h incubation period. Additionally, two other non-indole containing Nedd4 inhibitors reported in the literature were tested (**7**/NAB2 and **8**/heclin) over a range of concentrations and significantly inhibited Nedd4 (Supplementary Fig. 5)^31,32^.

**Figure 5:**
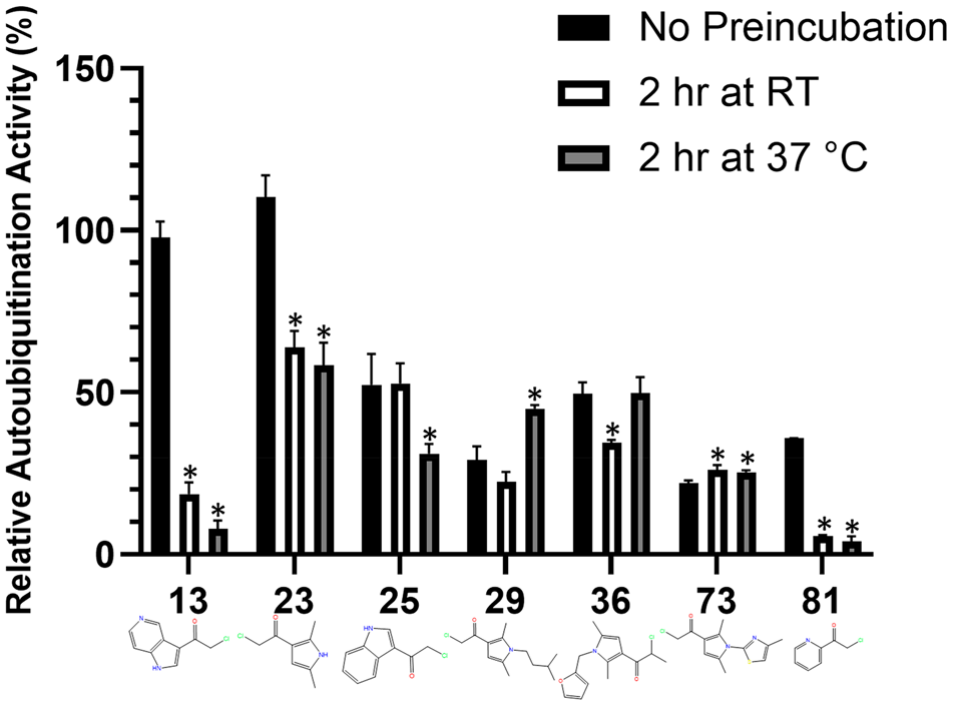
Effects of compound preincubation on the potency of inhibitors. Activities measured by difference in emission ratio at 528/485 nm between reactions with and without enzyme, are presented relative to the uninhibited assay in 1% DMSO (100% activity). Assays were run with or without a 2 h preincubation in 200 µM of inhibitor prior to initiation of the auto-ubiquitination reaction. Auto-ubiquitination reactions were allowed to proceed for 1 h at 37 °C after the addition of 2 mM ATP-MgCl_2_. Data represent means of three reactions performed in separate microplate wells ± standard deviation. Data for each compound were analyzed and compared to the relative activity without the preincubation through a one-way ANOVA with Dunnett's post hoc test applied. When the inhibition associated with one of the 2 h incubation conditions is significantly different (p < 0.05) from the control without preincubation the data is labeled with a '*'.

All compounds from the first and second set were selected for further testing along with 4 compounds from the final set that demonstrated near complete Nedd4 inhibition (less than 10% remaining activity) at 200 µM (Fig. 3C). These 28 compounds were assayed over a range of concentrations prepared by serial dilution to determine the IC_50_ for each after 2 h incubations as described in material and methods. From these compounds, 4 inhibitors (**13**, **23**, **25** and **81**) were identified with apparent IC_50_ values of less than 60 µM (Fig. 4). To test the covalent nature of compound inhibition, the auto-ubiquitination assay was performed at 200 µM with and without a 2 h preincubation of Nedd4 with each of the 7 compounds at RT or 37 °C (Fig. 5). Of the compounds tested, only **13** and **23** did not demonstrate any inhibition relative to the DMSO control without the 2 h compound preincubation prior to initiation of the auto-ubiquitination reaction (Fig. 5). In these cases, both **13** and **23** caused significant Nedd4 inhibition when preincubated for 2 h at either RT or 37 °C. By contrast, preincubation of **25** at 37 °C demonstrated only a moderately higher inhibition (31% activity compared with 52% without preincubation). **29** and **73** demonstrated ~ 29 and 22% remaining activity without a 2 h preincubation, respectively. For **36**, 2 h preincubation 37 °C did not result in any changes compared to the assay lacking the preincubation period. **81** demonstrated markedly greater inhibition with the preincubation step (36% activity compared to ~ 5% activity after preincubations at either temperatures) (Fig. 5).

### Confirmation of the inhibition of polyubiquitination by Western Blot

**Figure 6:**
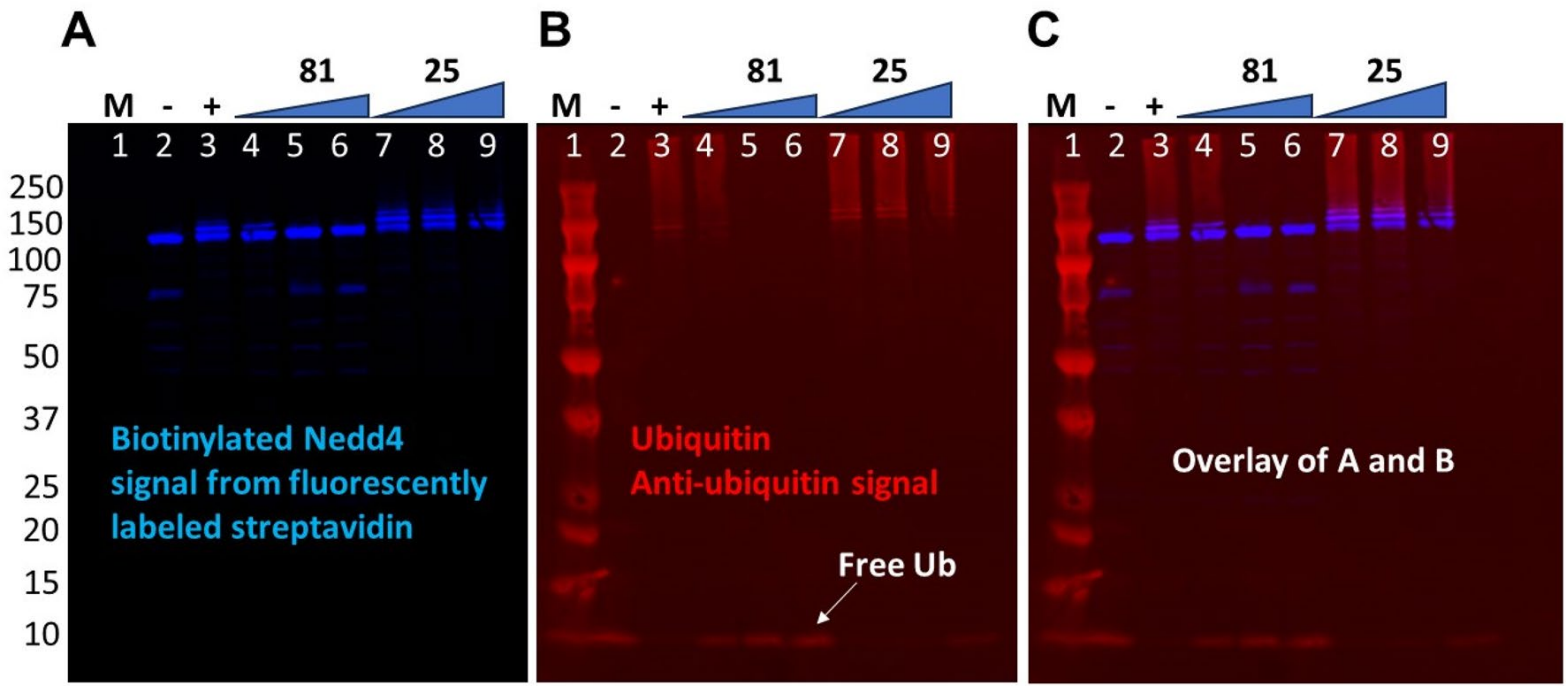
Confirmation of the inhibitory effect of compound **25** and **81** on Nedd4 auto-ubiquitination by Western Blot. Nedd4 auto-ubiquitination was monitored by (**A**) using fluorescently labeled streptavidin to visualize biotinylated full-length Nedd4, and (**B**) mouse anti-ubiquitin antibody and fluorescently labeled donkey anti-mouse secondary IgG antibody combination to detect ubiquitin. Anti-ubiquitin signal is shown in red and labeled streptavidin in blue. (**C**) The overlay of A and B is shown. Inhibition by two inhibitors **81** and **25** demonstrates differing potencies with **81** showing a complete loss of ubiquitination on the Nedd4 band at the two highest concentrations tested. However, **25** shows a modest loss of activity at 200 μM, most clearly indicated by the increase in the levels of free ubiquitin following the reaction in B and C. The samples in wells labeled 1 to 9 are as follows: (1) Protein molecular weight standard, (2) Reaction components minus ATP-MgCl_2_ (negative control), (3) Uninhibited reaction (positive control), (4) 10 µM **81**, (5) 50 µM **81**, (6) 100 µM **81**, (7) 50 µM **25**, (8) 100 µM **25**, (9) 200 µM **25**.

Immunoblotting for ubiquitin following the auto-ubiquitination reaction demonstrated clear confirmation of Nedd4 polyubiquitination (Fig. 6). Using a fluorescently labeled streptavidin to visualize biotinylated full-length Nedd4, the protein was ubiquitinated under the optimized conditions (positive control, Fig. 6A, lane 3) but not in the absence of ATP-MgCl_2_ (negative control, Fig. 6A, lane 2) as expected. Additionally, incubation of Nedd4 with increasing concentrations of **81** (10, 50 and 100 µM) demonstrated a clear inhibitory effect, with a complete loss of auto-ubiquitination at 50 and 100 μM, with the presence of only single Nedd4 band (lanes 5 and 6) similar to the negative control (lane 2). However, **25** with higher IC_50_ value (52 µM) than **81** (31 µM), showed a moderate effect at 200 µM (Fig. 6, lane 9). While inhibition by **25** and **81** could be observed with reduction of ubiquitinated Nedd4 bands at higher molecular weights (Nedd4 plus Ubiquitin molecules) in the blot, the inhibition of auto-ubiquitination could also be detected clearly (especially for **25** which is a weaker inhibitor) by the levels of remaining free ubiquitin proportional to the inhibitor concentration at the bottom of the blots (Fig. 6B,C). These results confirm the effect of both compounds on Nedd4 auto-ubiquitination.

### Mapping the sites of covalent modification

**Figure 7:**
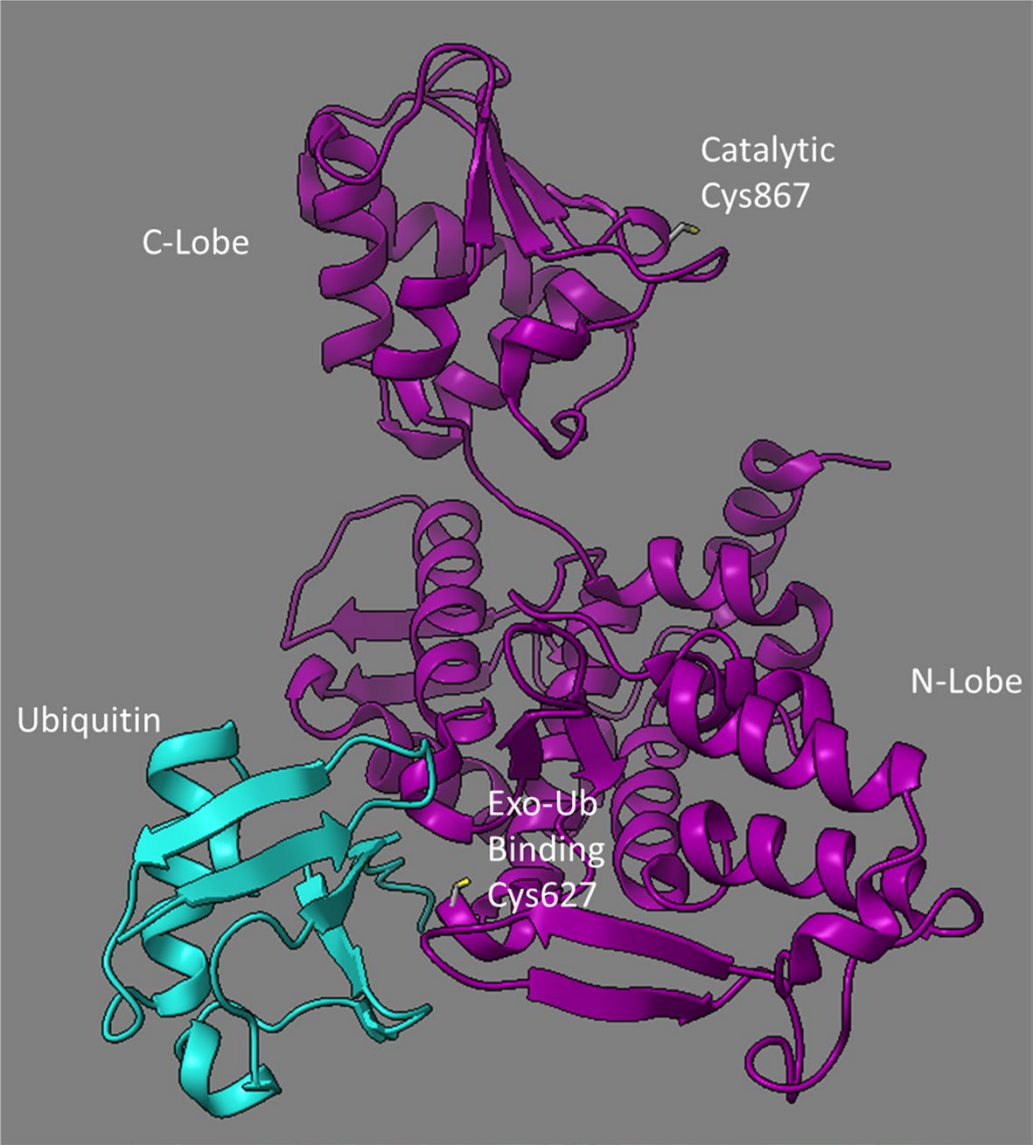
Visualization of the cysteine site locations in the Nedd4 HECT domain that are confirmed targets for adduction by compounds **25** and **81** in relation to the Ub exosite. Structure visualization of Nedd4-1 was performed using ChimeraX software on a solved crystal structure of human Nedd4 HECT domain bound to Ub in the Exo-Ub binding site (PDB: 2XBB).

To determine the covalent modification sites of selected compounds, full-length Nedd4 was incubated with each of the two most potent inhibitors (**25** and **81**) and samples were analyzed by MS/MS (Source Data 2). In total, three modified cysteine sites were identified through MS/MS for both compounds corresponding to Cys182, Cys627, and Cys867 in the full-length native Nedd4 protein. Of these cysteine adduction sites, only Cys627 and Cys867 are found within the HECT domain, and both of these sites have known roles in Nedd4 auto-ubiquitination activity. The Cys182, however, is located in a linker region between the N-terminal C2 and WW1 domains and is not known to have any mechanistic role in ubiquitination activity. **25** was only confirmed to adduct to Cys182 and Cys867, while **81** adducted to all three of the confirmed cysteine residues. The majority of detected **25** modifications occurred on Cys182 (41/95 peptides), while only 1 peptide was identified with the **25** adduct on Cys867 (Supplementary Table 4). In contrast, **81** adduction was reported relatively evenly across all three cysteines (Supplementary Table 4). Both the Cys627 and Cys867 residues are located in solvent-accessible regions of the HECT domain of Nedd4 (Fig. 7).

### Selectivity of top Nedd4 covalent inhibitors

To determine whether the identified compounds were pan-HECT domain inhibitors, compounds **13**, **23**, **25**, and **81** were tested for auto-ubiquitination inhibition of WWP1 and WWP2. The relative degrees of auto-ubiquitination of His-tagged WWP1 and WWP2 with and without incubation with these 4 compounds was measured using a similar Tb-anti-His-Ab-based TR-FRET assay (Supplementary Table 2).

**Figure 8:**
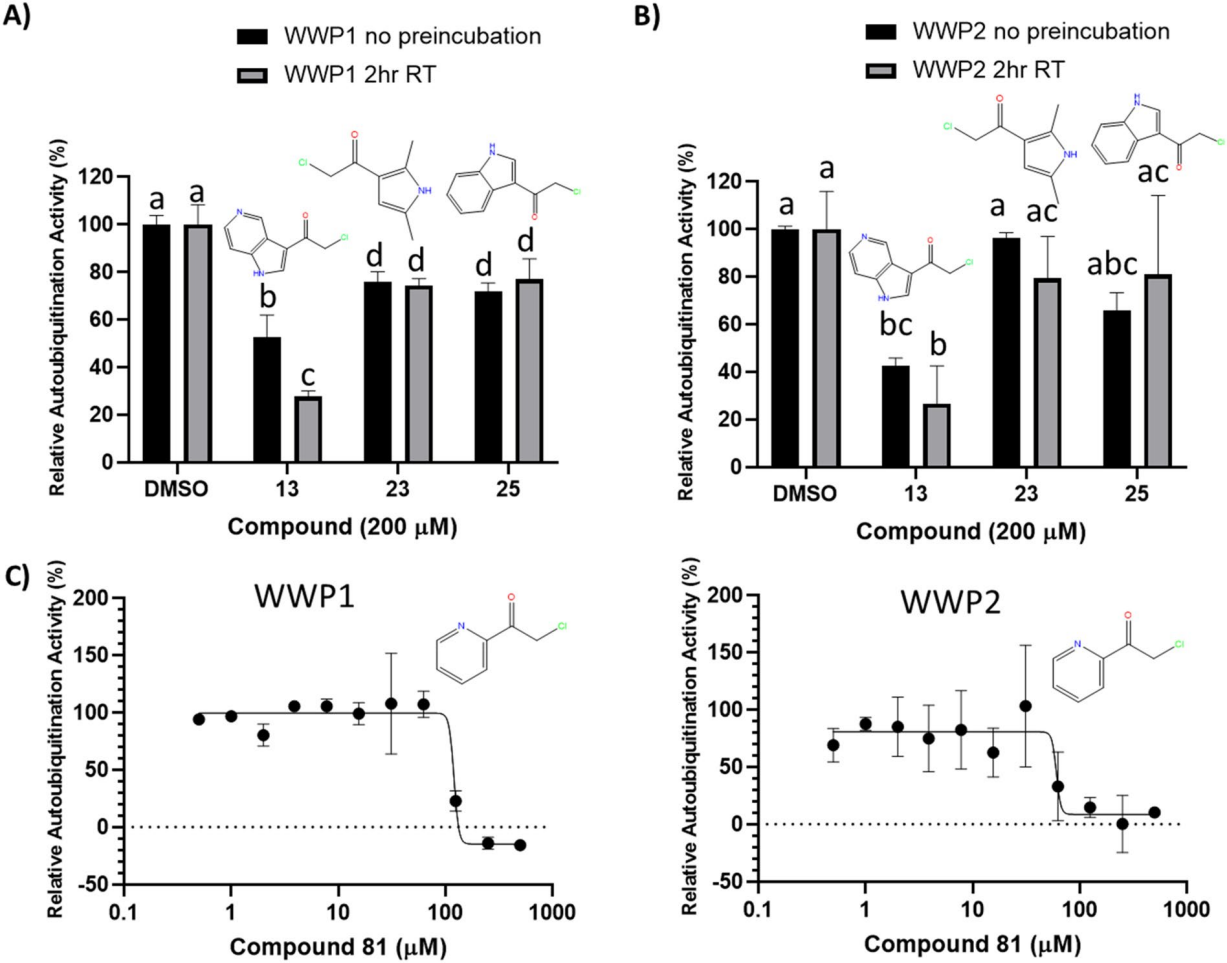
Auto-ubiquitination inhibition of the Nedd4-related HECT domain E3 ubiquitin ligases, WWP1 and WWP2, by Nedd4 inhibitors. (**A**) Comparing the effects of three Nedd4 inhibitors (**13**, **23** and **25**) with and without a 2 h RT incubation at 200 µM on the auto-ubiquitination activity of WWP1. Shared letters (a, b, c, d) between bars indicate that the null hypothesis cannot be rejected between two or more parameters by one-way ANOVA with Tukey's post-hoc test applied (p > 0.05). For example, the percent activities in the presence of compounds 23 and 25 with and without 2 h preincubation are not significantly different (all designated by letter “d”) (**B**) The inhibitory effects of the same Nedd4 inhibitors under the same conditions as in **A**) on the auto-ubiquitination activity of the WWP2. Letters above bars represent the same as in (**A**). (**C**) Plotting auto-ubiquitination activity of WWP1 (left) and WWP2 (right) against increasing concentrations of **81** (the most potent Nedd4 inhibitor discovered through the screen) following 2 h incubation at room temperature demonstrates clear inhibition of WWP1 and WWP2. All auto-ubiquitination reactions were performed in triplicate with the data representing the average ± standard deviation. Auto-ubiquitination activities are presented as a percent of the TR-FRET signal of the uninhibited reaction (DMSO).

**23** and **25** did not have any significant impact on WWP2 activity relative to the control at a concentration of 200 µM with or without preincubation and resulted in only a slight reduction in WWP1 activity (~ 25% inhibition), indicating selectivity of these compounds. In contrast, both **13** and **81** inhibited all three HECT E3 ligases and may be pan-HECT domain inhibitors (Fig. 8). The presence of 200 µM **13** demonstrated 47% inhibition of WWP1 auto-ubiquitination activity relative to the DMSO control without preincubation. Adding a 2 h RT **13** preincubation step to the assay yielded an additional reduction in WWP1 activity (72% inhibition) (Fig. 8A). Similarly, **13** demonstrated inhibition of WWP2 activity with 58% inhibition without the preincubation step and 73% inhibition with the 2 h RT preincubation (Fig. 8B). The Nedd4 inhibitor with the lowest IC_50_ (**81**) was assayed in dose–response for WWP1 and WWP2 auto-ubiquitination inhibition. Incubation of enzymes with **81** resulted in a complete and 85% loss of TR-FRET signal for WWP1 and WWP2 at concentrations above 125 µM, respectively (Fig. 8C).

### Inhibitory role of confirmed cysteine adduct sites

**Figure 9:**
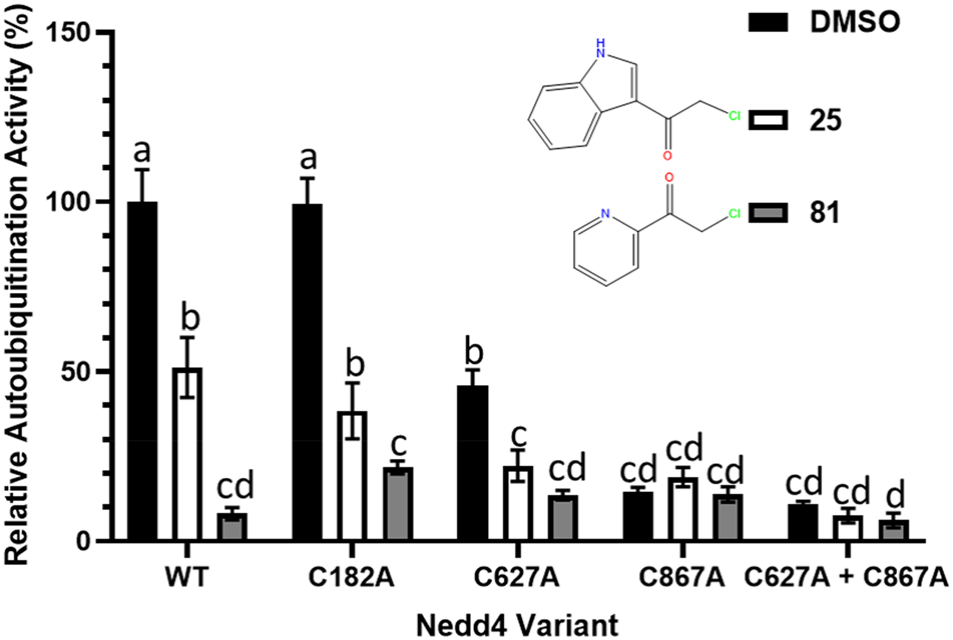
Effects of the two covalent inhibitors on the auto-ubiquitination activity of full-length wild type (WT) and mutant Nedd4 variants with MS confirmed cysteine adduction sites replaced with alanine. All assays were carried out in triplicate with the data representing the mean with standard deviation. Shared letters above bars (a, b, c, d) represent the null hypothesis cannot be rejected at the p < 0.05 level for any two parameters by one-way ANOVA with Tukey's post-hoc test. All wells were incubated first for 2 h at RT in 200 µM of the indicated compound dissolved in 1% DMSO or in just 1% DMSO. Auto-ubiquitination reactions were allowed to proceed for 1 h at 37 °C and then read through the TR-FRET assay described in the methods. All data are presented as percentages of the uninhibited (DMSO) wild type Nedd4 TR-FRET signal.

Since the Nedd4 inhibitors **25** and **81** have multiple confirmed cysteine adduction sites, Nedd4 variants with each modified cysteine substituted with alanine were purified to determine elimination of which cysteine residue may contribute to inactivation of the auto-ubiquitination, revealing the mechanism of inhibition of these two compounds. These Nedd4 mutants were designated C182A, C627A, and C867A. Additionally, a double mutant Nedd4 variant missing both cysteines located within the HECT domain (C627A + C867A) was also purified. The auto-ubiquitination activity of C182A was similar to the full-length wild-type (WT) Nedd4 (Fig. 9). In contrast, Nedd4 C876A demonstrated the greatest activity loss with only 15% activity compared to the wild type when incubated in 1% DMSO. C627A was moderately less active and demonstrated 46% of the WT Nedd4 activity under the same conditions. The double mutant (C627A + C867A) demonstrated the lowest TR-FRET signal (11% of the WT), although this was not significantly different from C867A.

To determine the role of the confirmed cysteine adduction sites of **25** and **81** in Nedd4 inhibition, the cysteine site mutants were subjected to incubation with these compounds and then assayed for auto-ubiquitination activity. Incubating C182A with **25** and **81** resulted in similar inhibition patterns compared to when WT Nedd4 was incubated with the respective compounds. Specifically, C182A incubation with **25** retained 49% activity relative to the DMSO control and was statistically indistinguishable from WT Nedd4, which retained 51% activity under the same conditions (Fig. 8). WT Nedd4 and C182A incubation with **81** resulted in a significantly lower TR-FRET response than incubation with **25**. **25** and **81** incubations also seem to have resulted in similar changes in the activity of C627A compared to WT Nedd4, with a 49% and 30% retention of uninhibited C627A activity for each inhibitor, respectively. However, these differences were not statistically distinguishable, potentially due to the mutant’s low activity, resulting in a higher relative error between replicates when compared to the WT enzyme. No significant differences were observed in the C867A or C627A + C867A mutants due to the Cys867 residue being essential for HECT domain catalytic activity.

## Discussion

E3 ubiquitin ligases are attractive targets for drug development within the proteasomal degradation pathway owing to the relatively specific downstream functions of individual E3 ligases compared to E1 and E2 enzymes, and thus inhibition of E3 ligases causes more targeted cellular effects. Accordingly, numerous techniques for assessing the activity of E3 ubiquitin ligases have been developed, with a variety of fluorescence-based techniques being described owing to the high sensitivity afforded by these techniques^33,34^. The present study describes a TR-FRET assay approach for the identification of inhibitors of Nedd4 auto-ubiquitination from a covalent fragment library.

Based on the current understanding of Nedd4 function, mutants lacking structural features known to regulate auto-ubiquitination activity were generated to validate the TR-FRET assay as a tool for probing Nedd4 activity. Nedd4 activity is intrinsically regulated through the effects of an inhibitory C2 domain located near the N-terminal of the protein^9^. A truncated Nedd4 variant (153–900) lacking the inhibitory C2 domain demonstrated increased auto-ubiquitination activity, as expected when compared to the WT Nedd4 (Fig. 2A). The linker peptide (225–244) C-terminal to the WW1 domain has intrinsic Nedd4 inhibitory properties, and it was expected from previous studies that deletion of this region in addition to the loss of the C2 domain would cause an increase in auto-ubiquitination activity compared to the variant lacking the C2 domain only^10^. Surprisingly, the mutant lacking the linker peptide showed the same level of activity as the truncated protein lacking the C2 domain in contrast to the previous work on the subject, which suggested that these two regions have non-redundant mechanisms of Nedd4 autoinhibition^10^. Nedd4 cysteine mutants were used as a tool to further validate the mechanism of inhibition of the compounds. As expected, replacing the catalytic Cys867 residue within the HECT domain with an alanine residue resulted in an almost complete loss of TR-FRET signal, and replacing the Ub exosite Cys627 involved in enhancing Ub chain elongation demonstrated a 50% loss of TR-FRET signal relative to the WT Nedd4 (Fig. 9)^35^. Taken together, these observations confirm that the TR-FRET signal generated from the method described here is an accurate metric for assessing Nedd4 activity in vitro.

The TR-FRET assay was used to investigate known inhibitors of Nedd4 and several analogs for inhibitors of Nedd4 auto-ubiquitination. The structural analogs of the Nedd4 indole-based inhibitor I3C (**5**) was used as a comparison point for potential Nedd4 inhibitors that would be discovered in the covalent library screen. Although an accurate IC_50_ for this compound could not be calculated based on the data here, as higher concentrations would be needed, this compound has been previously reported to have an IC_50_ of 284 µM^25^. Consistently, two of the inhibitors (**13** and **25**) identified through the screening of the covalent library contained indole groups and have a similar structure to I3C. **13** and **25** are almost completely structurally identical to each other except for **13** having a nitrogen substituted in the 6^th^ position of the bicyclic indole ring (Fig. 4). These screening hits showed promise since both have much lower apparent IC_50_ values following the 2 h RT incubation (both around 52 µM) compared to the previously reported I3C IC_50_ (284 µM), suggesting that covalent adduction improves the inhibitory potency of the compound. Additionally, both **13** and **25** exhibited greater maximum inhibitions at their highest concentrations (**13**: 81% and **25**: 97.5% inhibition) than I3C (**5**: 33%) despite the highest concentration of **13** and **25** being only 300 µM compared to 500 µM for **5** (I3C) (Fig. 3A,B). By comparison, 1-benzyl-I3C, an I3C derivative, has been previously shown to have an IC_50_ of 12.3 µM measured through an ELISA-based assay of Nedd4 activity^25^. Expanding the search for inhibitors to compounds more structurally dissimilar to I3C revealed several other inhibitors that were not closely related to I3C. The most potent of all compounds discovered in the screening was 81, with an apparent IC_50_ value of 31 µM. This compound has a comparatively simple structure, consisting of a pyridine heterocycle with a nitrogen located one position away from the electrophile substituent group. **23** also demonstrated significant Nedd4 inhibition and represents a departure from the indole-containing compounds. **23** lacks an indole group, but rather contains a five membered aromatic heterocycle with two methyl groups. The inhibitory mechanism of this compound is uncharacterized, but inhibition of Nedd4 followed a time-dependent trend, strongly suggesting covalent binding (Fig. 5). Notably, several other hits from the screen are derivatives of 23 (19, 20, 21, 29, 36, 73), each bearing a different group on the nitrogen atom. However, **23** was the most potent inhibitor of this group, suggesting that adding more bulky groups to the nitrogen hinders Nedd4 inhibition.

Tandem mass spectrometry mapped the adduction sites of the two most potent inhibitors, **25** and **81**, on three of the five cysteine residues within the full-length Nedd4 (Supplementary Fig. 6). However, **25** demonstrated lower rates of adduction to the catalytic Cys867 residue and no peptides were found with modification at Cys627. The lack of adducts to the Cys627 residue by **25** is surprising considering **25** is a close analog of the previously reported indole class of Nedd4 inhibitors that are predicted to interact in the Ub exosite binding pocket in close proximity to the non-catalytic Cys627^36^. It is likely that Cys867 and Cys182 react preferentially with the electrophilic reactive group on these library compounds making effective covalent targeting of indole-based inhibitors to this exosite pocket dependent on additional derivatization from the I3C inhibitor core structure to more preferentially bind this site^25,27^. Indeed, maximal inhibition of **25** is close to 100%, which cannot be explained by binding to Cys627 alone since mutation of this residue to alanine resulted in only a 50% inhibition of Nedd4 autoubiquitination activity (Fig. 8). By contrast, **4** (a derivative of I3C), which was previously reported as a Nedd4 inhibitor specifically recognizing the Cys627 site over the Cys867 site^27^, had maximum auto-ubiquitination inhibition of approximately 50% through TR-FRET assay (Figs. 3, 4).

Of the three cysteine sites found to be modified by the compounds, Cys182 is the only one that is not located within the HECT domain and does not have a characterized role in promoting the catalytic transfer of ubiquitin to Nedd4. Since Cys182 is positioned in an unstructured region N-terminal to the WW1 domain (Fig. 1), it is predicted to be highly solvent-exposed, which may explain the high degree of modification at Cys182 for both **25** and **81**. Based on the crystal structure of Nedd4, both Cys627 and Cys867 within the HECT domain are also solvent accessible^27^. Indeed, both Cys627 and Cys867 are known to have important roles in promoting Nedd4 auto-ubiquitination. Cys867 is the active site cysteine that catalyzes the transfer of Ub from the E2 to the E3 (Fig. 7) and therefore, modifying Cys867 with **25** or **81** should entirely block the transthioesterification reaction and prevent Ub transfer from the E2 to Nedd4. Point mutants lacking the Cys867 catalytic residue were assayed for auto-ubiquitination activity and demonstrated only 15% retention of activity. This unexpected TR-FRET signal above baseline may be due to Ub-FITC transiently and non-covalently binding in the active site or Ub exosite, since this mutant is catalytically inactive. Therefore, the TR-FRET signal yielded by C867A Nedd4 should approximate the signal obtained for WT Nedd4 that has undergone complete adduction at the catalytic site. Conversely, Cys627 is a non-catalytic cysteine in the Ub exosite. The Ub exosite within the HECT domain is located on the N-lobe and plays a regulatory function by binding to a Ub molecule to promote ubiquitination. It is currently thought that Ub binding at the exosite is responsible for promoting processivity of poly-Ub chain elongation and is a known target for Nedd4 inhibition^27,37^. The partial loss of autoubiquitination activity when lacking the Cys627, a residue associated with enhancing chain elongation on a substrate molecule, is consistent with a previous study which demonstrated that Nedd4 is capable of K29 auto-polyubiquitination^38^. Thus, targeted modification of Cys627 through compound adduction should inhibit but not completely abolish Nedd4 auto-ubiquitination activity.

Auto-ubiquitination assays on mutant Nedd4 variants in the presence of **25** and **81** were performed to better understand the role of these adductions in compound inhibition. The Cys182 residue identified through tandem mass spectrometry as a site of adduct formation for both **25** and **81** was of particular interest since this site is located far away from the HECT domain and does not have any previously established role in mediating ubiquitination^39^. Additionally, modification at Cys182 was the most common adduction site for **25** with 41/95 identified peptides bearing this adduct. However, assessing the auto-ubiquitination activity of C182A did not reveal any difference to the WT activity. This suggests that the Cys182 residue, despite being a common site of adduction for both compounds, is not relevant to the inhibition of Nedd4 activity. Rather, the mechanism of **25** mediated inhibition is likely through adduction to Cys867 since there is no observed adduction to the C627 Ub exosite. Additionally, the activity ratio of enzyme incubated in **25** versus the 1% DMSO control is approximately equal in both C627A and WT Nedd4. This indicates that the effects of **25** incubation on Nedd4 activity are unchanged when the Cys627 site is absent and supports the conclusion that the primary mechanism of **25** inhibition is through blocking the Cys867 residue.

Due to critical importance of Cys867 in Nedd4 activity, it is logical that screens selecting for the potent covalent inhibitors of Nedd4 activity would generate large numbers of hits that adduct to the Cys867 residue. The TR-FRET signal from WT and C182A Nedd4 incubated with **81** are not statistically different from the inactive C867A variant incubated in DMSO further suggesting that **81** adduction to Cys867 under these conditions is likely near completion. In contrast to the catalytic Cys867 residue, adduction to the Cys627 residue is not likely the main mode of inhibition of these compounds. The C627A variant has approximately half of the auto-ubiquitination activity as the WT in DMSO and therefore the maximal inhibition caused by adduction to this residue should yield approximately 50% activity in the WT enzyme. Therefore, the observation that **81** results in considerably more than 50% inhibition in the WT demonstrates that the primary mode of inhibition is through the blocking of the catalytic Cys867 site. Taken together, the results from these mutant Nedd4 studies help to demonstrate that inhibition by the top inhibitors discovered through the screen takes place predominantly through adduct formation to the Cys867 residue.

The Cys867 residue in Nedd4 forms the catalytic core of the HECT domain and is conserved in all E3 ubiquitin ligases in the Nedd4 family^40^. In contrast, Cys627 residue on the N-lobe of the HECT domain in Nedd4 is not conserved in other HECT containing E3 ligases such as WWP1 and WWP2 (Supplementary Fig. 6). Thus, compounds that only form adducts with Cys627 are not expected to result in specific pan-HECT domain inhibition^27^. However, the results of our study suggest that the main mode of both **25** and **81** Nedd4 inhibition is mediated through binding to Cys867 residue. The most effective Nedd4 inhibitors described here displayed mixed results in terms of their ability to inhibit the related WWP1 and WWP2 E3 ubiquitin ligases. **13** was noted to have a pronounced inhibitory effect on WWP1/2 activity despite being very structurally similar to **25** which exhibited minimal inhibition. **13** is identical to **25** except for an additional nitrogen atom incorporated into the six-membered aromatic ring of its indole group. Indole-3-carbinol (I3C), which has a similar structure to **13** has been identified as an inhibitor of WWP1 activity through its interactions with a binding pocket located on the N-terminal of the WWP1 HECT domain in a similar manner to Nedd4 inhibition^30^. However, the lack of a cysteine in the N-terminal Ub binding site for **13** adduction suggests that covalent inhibition of WWP1/2 occurs in the conserved catalytic cysteine site. **23** is notable in that it did not demonstrate any significant inhibition in WWP2 while displaying weak inhibition for WWP1. In contrast, Nedd4 demonstrated a retention of only 33% activity after incubation at 300 µM of **23**. Taken together, these results demonstrate that the initial screen of library compounds for Nedd4 inhibitors uncovered molecules varying in inhibitory specificity, with two confirmed to preferentially inhibit Nedd4 over WWP1/2 and another two functioning as pan-HECT domain inhibitors.

## Conclusion

The present study reports the development of a TR-FRET assay for assessing in vitro auto-ubiquitination activity of Nedd4 E3 ligase and its suitability for screening potential small molecule inhibitors. Through screening a collection of selected compounds, we identified several covalent inhibitors of Nedd4 auto-ubiquitination and determined the target residues on Nedd4 through mass spectrometry. Our data elucidate the mechanism of inhibition of these inhibitors and supports the critical involvement of catalytic Cys867, and potentially a role for Cys627 in Nedd4 auto-polyubiquitination. Selectivity of the top compounds were tested on the two Nedd4 closely related members, WWP1 and WWP2, which demonstrated that the two most potent Nedd4 inhibitors discovered through the Nedd4 screen showed comparatively low inhibition for either of the other family members, indicating selectivity. Taken together, these results could aid future studies at exploring the design and refinement of more potent and selective inhibitors for Nedd4 and related HECT domain ubiquitin ligases.

### Supplementary Information

Supplementary Information 1.

Supplementary Information 2.

Supplementary Information 3.

## Data Availability

All supporting data related to this manuscript are available through the Supplementary files and source files.
