# Supplementary material for: Discovery of Nedd4 auto-ubiquitination inhibitors
Source: Sci Rep. 2023 Sep 25;13:16057. doi: 10.1038/s41598-023-42997-z (PMC10520017; doi:10.1038/s41598-023-42997-z)
Supplement: Supplementary file 1 — Supplementary Information 1. [file 41598_2023_42997_MOESM1_ESM.pdf]

Supplementary Materials

Discovery of Nedd4 auto-ubiquitination inhibitors

Darren Yong[1†], Stuart R. Green[1†], Pegah Ghiabi[1], Vijayaratnam Santhakumar[1], Masoud Vedadi[1,2,3*]

[1]Structural Genomics Consortium, University of Toronto, Toronto, Ontario, M5G 1L7, Canada

[2]Department of Pharmacology and Toxicology, University of Toronto, Toronto, Ontario, M5S 1A8, Canada

[3]Drug Discovery Program, Ontario Institute for Cancer Research, Toronto, Ontario, Canada.

[†] Authors contributed equally to this work

*To whom correspondence should be addressed:
Masoud Vedadi; Tel.: 416-432-1980; E-mail: m.vedadi@utoronto.ca

Table of Contents

	Page
**Table 1. Fluorophore information for components of Nedd4 TR-FRET assay**	**3**
**Table 2. TR-FRET assay components for Nedd4, WWP1, and WWP2**	**4**
**Table 3. The list of all 79 compounds, SMILES and potencies.**	**5**
**Table 4. Identification of 25 and 81 Cys adduction sites through MS/MS**	**8**
**Figure 1. Optimization of assay buffer components for Nedd4 TR-FRET assay**	**9**
**Figure 2. Optimization of NP-40 concentration for Tb fluorescence**	**10**
**Figure 3. Optimization of protein concentrations for Nedd4 TR-FRET assay**	**11**
**Figure 4. Z′-factor determination and increase in TR-FRET signal over time**	**12**
**Figure 5. Nedd4 inhibition by known inhibitors measured by TR-FRET assays**	**13**
**Figure 6. Sequence alignment of Nedd4 and WWP1/2 overlayed with confirmed 25 and 81 Nedd4 adduction sites**	**14**
**Supplementary Materials and Methods**	**16**

Table S1. Fluorophore information for the Tb-SA and the Ub-FITC used in the Nedd4 assay.

	LanthaScreen™ Elite Tb-SA	FITC labeled ubiquitin
Vendor	Thermo Scientific	Thermo Scientific
Catalog number	PV3965	PV4378
Label/Dye	Terbium	FITC (Fluorescein)
Excitation (nm)	~340	~485
Emission (nm)	~485	~528

Table S2. Standard autoubiquitination TR-FRET assay conditions for auto-ubiquitination assays for HECT domain E3 ligases. In Nedd4 assay, we used FITC-labelled ubiquitin and C-terminal biotin-labelled Nedd4 protein conjugated to Tb-SA for generating the TR-FRET signal. For selectivity assays, we used C-terminal His-tagged WWP1 and WWP2 bound to Tb-labelled anti-His-tag antibody and FITC-labelled ubiquitin.

**Nedd4**		**WWP1**		**WWP2**	
E1 (uba1)	50 nM	E1 (uba1)	120 nM	E1 (uba1)	50 nM
E2 (UbcH5a)	125 nM	E2 (UbcH7)	2000 nM	E2 (UbcH7)	400 nM
E3 (Nedd4)	130 nM	E3 (WWP1)	100 nM	E3 (WWP2)	100 nM
Ub-FITC	300 nM	Ub-FITC	440 nM	Ub-FITC	300 nM
Tb-SA	50 nM	AntiHis-Tb	16.5 nM	AntiHis-Tb	10 nM
NaCl	150 mM	NaCl	0 mM	NaCl	0 mM
HEPES pH 7.5	20 mM	HEPES pH 7.5	50 mM	HEPES pH 7.5	50 mM
TCEP	0.1 mM	TCEP	0.1 mM	TCEP	0.1 mM
DMSO	1 %	DMSO	1 %	DMSO	1 %
NP-40	0.00063 %	NP-40	0.0002%	NP-40	0.0002%
$MgCl_2$	2 mM	$MgCl_2$	2 mM	$MgCl_2$	2 mM
ATP	2 mM	ATP	2 mM	ATP	2 mM

Table 3. The list of all 79 compounds, SMILES and potencies.

Compounds from Figure 3A (screened at 500 μM), and Figure 4 (IC_{50} determination)

Compound	Catalog Name	SMILES	IC50 (μM)	MW (g/mol)	Supplier
1	EN300-04394	C(C(c1c[nH]c2ccccc12)=O)[Cl]	91	193.633	Enamine
2	Z1255433268	CC(=C)C(c1c[nH]c2ccccc12)=O	280	185.226	Enamine
3	QD-1755	COCc1c[nH;v2]c2ccccc12	NA	160.196	Combi-Blocks
4	EN300-248299	Cc1c(C(=NC/C=C/C(=O)OC)[O;v1])c2cc(ccc2n1C1CCCC1)OC	NA	369.441	Enamine
5	EN300-18594	C(c1c[nH;v2]c2ccccc12)O	NA	146.169	Enamine
6	EN300-1709160	Cn1cc(C(C[Cl])=O)c2ccccc12	170	207.66	Enamine

Compounds from Figure 3B (screened at 300 μM), and Figure 4 (IC_{50} determination)

Compound	Catalog Name	SMILES	IC50 (μM)	MW (g/mol)	Supplier
9	Z56867174	Cn1c(c2ccccc2)c(C(C[Cl])=O)c2ccccc12	NA	283.758	Enamine
10	Z89283959	C(C(c1c2ccccc2[nH]c1c1ccc(cc1)F)=O)[Cl]	NA	287.721	Enamine
11	Z1157651610	COC(c1ccc2c(c1)c(c[nH]2)C(C[Cl])=O)=O	100	251.669	Enamine
12	Z57014416	C(C(c1c2ccccc2[nH]c1c1ccccc1)=O)[Cl]	NA	269.731	Enamine
13	Z1783110847	C(C(c1c[nH]c2ccncc12)=O)[Cl]	52	194.621	Enamine
14	Z1202190152	Cc1c(C(C[Cl])=O)c2cc(ccc2[nH]1)OC	NA	237.686	Enamine
15	Z385443974	Cc1cccc2c1c(c[nH]2)C(C[Cl])=O	NA	207.66	Enamine
16	Z1160902697	C(C(c1c[nH]c2ccc(cc12)F)=O)[Cl]	NA	211.623	Enamine
17	Z56914850	CCc1cccc2c(c[nH]c12)C(C[Cl])=O	NA	221.687	Enamine
18	Z56877842	CCn1c(c2ccccc2)c(C(C[Cl])=O)c2ccccc12	NA	297.785	Enamine
19	Z56953088	Cc1cc(C(C[Cl])=O)c(C)n1Cc1ccccc1	180	261.752	Enamine
20	Z56794771	Cc1cc(C(C[Cl])=O)c(C)n1c1ccccc1	240	247.725	Enamine
21	Z56876399	Cc1cc(C(C[Cl])=O)c(C)n1CCOC	140	229.707	Enamine
22	Z1415953981	CC(C)Oc1ccc2c(c1)c(c[nH]2)C(C[Cl])=O	NA	251.713	Enamine
23	Z235542844	Cc1cc(C(C[Cl])=O)c(C)[nH]1	56	171.627	Enamine
24	Z56347313	Cn1ccc(c1)C(C[Cl])=O	115	157.6	Enamine
25	Z56886524	C(C(c1c[nH]c2ccccc12)=O)[Cl]	52	193.633	Enamine
26	Z359384108	Cn1cc(cc1C(N)=O)C(C[Cl])=O	NA	200.625	Enamine

Compounds from Figure 3C (screened at 500 μM), and Figure 4 (IC_{50} determination)

Compound	Catalog Name	SMILES	IC50 (µM)	MW (g/mol)	Supplier
27	Z56893163	Cc1cc(C(C[Cl])=O)c(C)n1Cc1cccs1	NT	267.781	Enamine
28	Z126620916	Cc1c(C(C[Cl])=O)c(C(=O)OC)c(C)n1C1CC1	NT	269.728	Enamine
29	Z56933001	CC(C)CCn1c(C)cc(C(C[Cl])=O)c1C	90	241.762	Enamine
30	Z56989551	Cc1cc(C)cc(c1)n1c(C)cc(C(C[Cl])=O)c1C	NT	275.779	Enamine
31	Z56995187	Cc1cc(C(C[Cl])=O)c(C)n1c1ccc(cc1)OC(F)(F)F	NT	331.721	Enamine
32	Z56995195	Cc1cc(C(C[Cl])=O)c(C)n1c1cc(cc(c1)[Cl])[Cl]	NT	316.615	Enamine
33	Z56347377	Cc1cc(C(C[Cl])=O)c(C)n1c1ccc(c(c1)[Cl])OC(F)F	NT	348.176	Enamine
34	Z55928962	CC(C(c1cc(C)n(c2ccccc2)c1C)=O)[Cl]	NT	261.752	Enamine
35	Z56995184	Cc1ccc(cc1F)n1c(C)cc(C(C[Cl])=O)c1C	NT	279.742	Enamine
36	Z55993049	CC(C(c1cc(C)n(Cc2ccco2)c1C)=O)[Cl]	110	265.74	Enamine
37	Z57008881	Cc1cc(C(C[Cl])=O)c(C)n1C(c1ccccc1)c1ccccc1	NT	337.85	Enamine
38	Z57000587	CCOc1ccc(cc1)n1c(C)cc(C(C[Cl])=O)c1C	NT	291.778	Enamine
39	Z221592478	CCOC(c1c(C)c(C(C[Cl])=O)c(C)[nH]1)=O	NT	243.69	Enamine
40	Z56862262	CCOC(c1c(C(C[Cl])=O)c(C)[nH]c1C)=O	NT	243.69	Enamine
41	Z56938159	CC(C(c1cc(C)n(c2ccc(cc2)OC(F)F)c1C)=O)[Cl]	NT	327.758	Enamine
42	Z3211927495	Cc1coc(C)c1C(C[Cl])=O	NT	172.611	Enamine
43	Z56877861	Cc1cc(C(C[Cl])=O)c(C)n1c1ccc(cc1)OC	NT	277.751	Enamine
44	Z56989550	CC(C)c1ccc(cc1)n1c(C)cc(C(C[Cl])=O)c1C	NT	289.806	Enamine
45	Z1762860628	Cc1cc(co1)C(C[Cl])=O	NT	158.584	Enamine
46	Z56924433	Cc1cc(C(C[Cl])=O)c(C)n1CCc1ccc(cc1)F	NT	293.769	Enamine
47	Z57033952	Cc1cc(C(C[Cl])=O)c(C)n1c1ccc(cc1)C(F)(F)F	NT	315.722	Enamine
48	Z56948394	Cc1cc(C(C[Cl])=O)c(C)n1c1ccc2c(c1)OCO2	NT	291.734	Enamine
49	Z56347520	Cc1cc(C(C[Cl])=O)c(C)n1c1ccc(cc1)S(N)(=O)=O	NT	326.805	Enamine
50	Z57035557	Cc1cc(C(C[Cl])=O)c(C)n1c1cccc(c1)[N+]([O-])=O	NT	292.722	Enamine
51	Z119981164	Cc1cc(C(C[Cl])=O)c(C)n1c1ccccn1	NT	248.713	Enamine
52	Z85926085	Cc1cc(C(C[Cl])=O)c(C)n1CCc1cccs1	NT	281.808	Enamine
53	Z56979405	Cc1cc(C(C[Cl])=O)c(C)n1c1cccc(c1)SC	NT	293.819	Enamine
54	Z56924552	Cc1cc(C(C[Cl])=O)c(C)n1CCc1ccc(cc1)[Cl]	NT	310.224	Enamine
55	Z94599348	Cc1cc(C(C[Cl])=O)c(C)n1CC(N)=O	NT	228.679	Enamine
56	Z56926562	Cc1cc(C(C[Cl])=O)c(C)n1Cc1ccccc1[Cl]	NT	296.197	Enamine
57	Z57033953	Cc1cc(C(C[Cl])=O)c(C)n1Cc1ccc(cc1)F	NT	279.742	Enamine
58	Z57014409	CC(c1ccccc1)n1c(C)cc(C(C[Cl])=O)c1C	NT	275.779	Enamine
59	Z56797551	Cc1cc(C(C[Cl])=O)c(C)n1CCc1ccccc1	NT	275.779	Enamine
60	Z56979286	Cc1cc(C(C[Cl])=O)c(C)n1c1cc(C)on1	NT	252.701	Enamine
61	Z56893168	Cc1cc(C(C[Cl])=O)c(C)n1c1ccc(c(c1)[Cl])[Cl]	NT	316.615	Enamine
62	Z56917411	Cc1cc(C(C[Cl])=O)c(C)n1c1ccc(cc1)[Cl]	NT	282.17	Enamine
63	Z56794691	Cc1cc(C(C[Cl])=O)c(C)n1c1ccc(c(c1)C(F)(F)F)[Cl]	NT	350.167	Enamine
64	Z57033950	Cc1cc(C(C[Cl])=O)c(C)n1c1cccc(c1)[Cl]	NT	282.17	Enamine
65	Z228588864	Cc1ccccc1n1c(C)cc(C(C[Cl])=O)c1C	NT	261.752	Enamine
66	Z56794771	Cc1cc(C(C[Cl])=O)c(C)n1c1ccccc1	NT	247.725	Enamine
67	Z228589028	Cc1cc(C(C[Cl])=O)c(C)n1c1ccc(cc1)C(C)(C)C	NT	303.833	Enamine
68	Z147527710	CC(C)n1c(C)c(C(C[Cl])=O)c(C(=O)OC)c1C	NT	271.744	Enamine
69	Z90664854	Cc1cc(C(C[Cl])=O)c(C)n1c1ccon1	NT	238.674	Enamine
70	Z1354601779	Cc1cccc(c1)n1cc(C(C[Cl])=O)nn1	NT	235.674	Enamine
71	Z56877841	Cc1cc(C(C[Cl])=O)c(C)n1CC1CCCO1	NT	255.745	Enamine
72	Z57011402	Cc1cc(C(C[Cl])=O)c(C)n1C1CCS(C1)(=O)=O	NT	289.784	Enamine
73	Z56347276	Cc1csc(n1)n1c(C)cc(C(C[Cl])=O)c1C	NA	268.769	Enamine
74	Z119967280	C1CCN(C1)C(c1cc(c[nH]1)C(C[Cl])=O)=O	NT	240.69	Enamine
75	Z1192340728	Cc1c(cc(C(N)=O)o1)C(C[Cl])=O	NT	201.609	Enamine

76	Z1171753358	Cc1c(cc(C(O)=O)o1)C(C[Cl])=O	NT	202.593	Enamine
77	Z228589230	Cc1cccc(c1)n1c(C)cc(C(C[Cl])=O)c1C	NT	261.752	Enamine
78	Z164679570	Cn1cc(cc1C(C([Cl])([Cl])[Cl])=O)C(C[Cl])=O	NT	302.972	Enamine
79	Z372795226	Cc1c(C(C[Cl])=O)c(C)on1	NT	173.599	Enamine
80	Z1205661058	C(C(c1ccoc1)=O)[Cl]	NT	144.557	Enamine
81	Z1205657985	C(C(c1ccccn1)=O)[Cl]	31	155.584	Enamine

Table S4. Identification of compound binding sites for compounds 25 and 81 through MS/MS following a 3 h incubation in two compounds of interest. Table gives the location of the modification and the compound responsible at the respective cysteine binding sites.

Catalog Number	Designation	Structure	Cysteine Site	Surrounding Sequence	Occurrence Among Observed Peptides
Z56886524	**25**	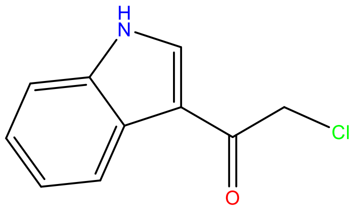	C182	DQODAACHLQQQQ	41/95
			C867	LPRAHTCFNRLDL	1/76
Z1205657985	**81**	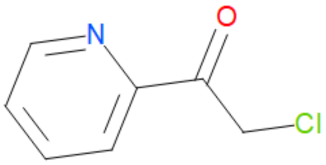	C182	DQODAACHLQQQQ	25/67
			C627	NPNSGLCNEDHLS	8/12
			C867	LPRAHTCFNRLDL	21/50

Figure S1. Optimization for Nedd4 TR-FRET autoubiquitination assay buffer components. A) NaCl, **B)** HEPES pH 7.5, **C)** DTT, **D)** TCEP, **E)** Triton X-100 and **F)** DMSO. All buffers contain 2 mM ATP-$MgCl_2$, NaCl, HEPES, DTT, TCEP, Triton X-100 and DMSO as indicated.

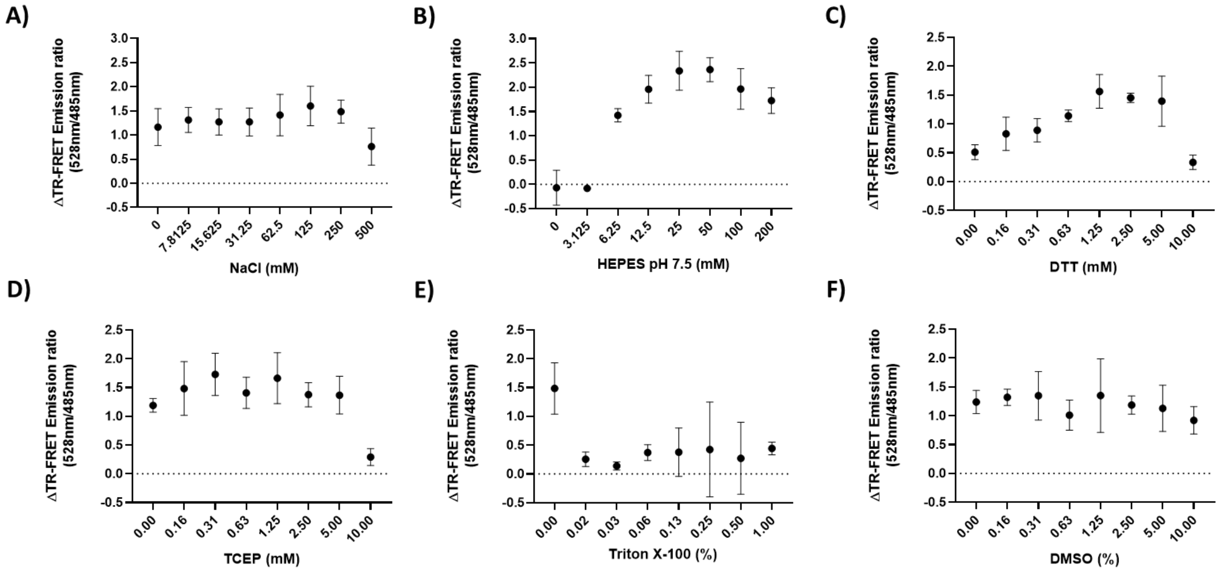

Figure S2. Optimization of NP-40 concentration for optimal Tb-SA time-resolved fluorescence. The effect of NP-40 on Tb-SA emission at 485 nm after excitation at 340 nm was tested at various incubation time ranging from 5 to 105 minutes at room temperature. All conditions contained 40 mM HEPES pH 7.5, 2 mM ATP-$MgCl_2$. All data points represent an average of three independent measurements ± standard deviation.

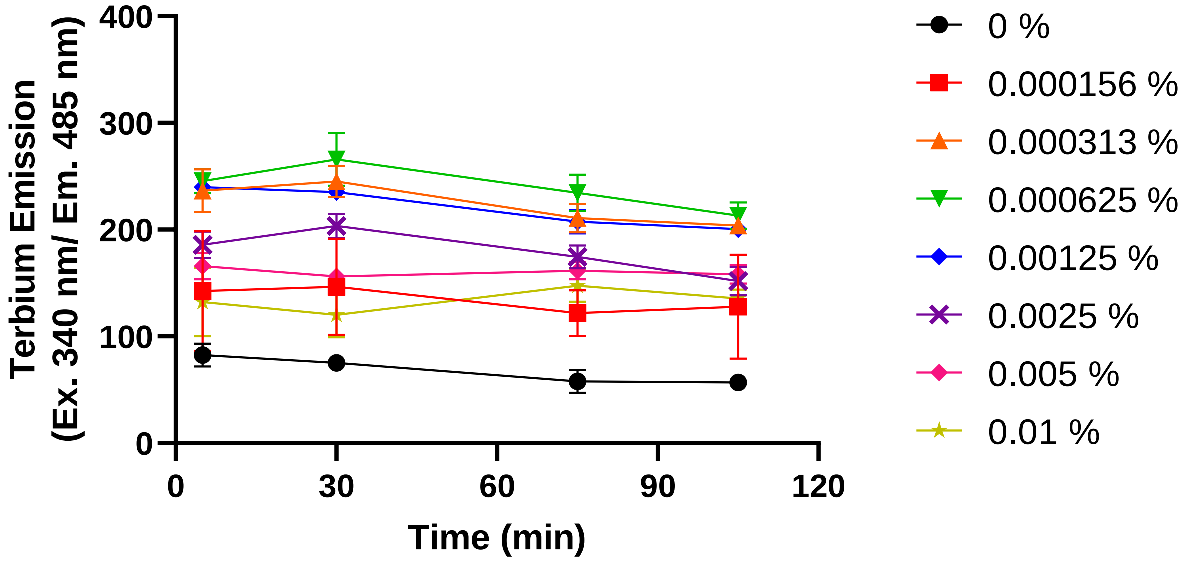

Figure S3. Optimization of protein concentrations in the TR-FRET assay. The optimum Nedd4 TR-FRET assay conditions was determined by varying the concentrations of **A)** E1, **B)** E2, **C)** E3, **D)** FITC-Ub, **E)** Tb-SA and **F)** Ub-WT. All buffers contain 150 mM NaCl, 20 mM HEPES pH 7.5, 0.00063 % NP-40, 2 mM ATP-$MgCl_2$ and the indicated concentration of proteins.

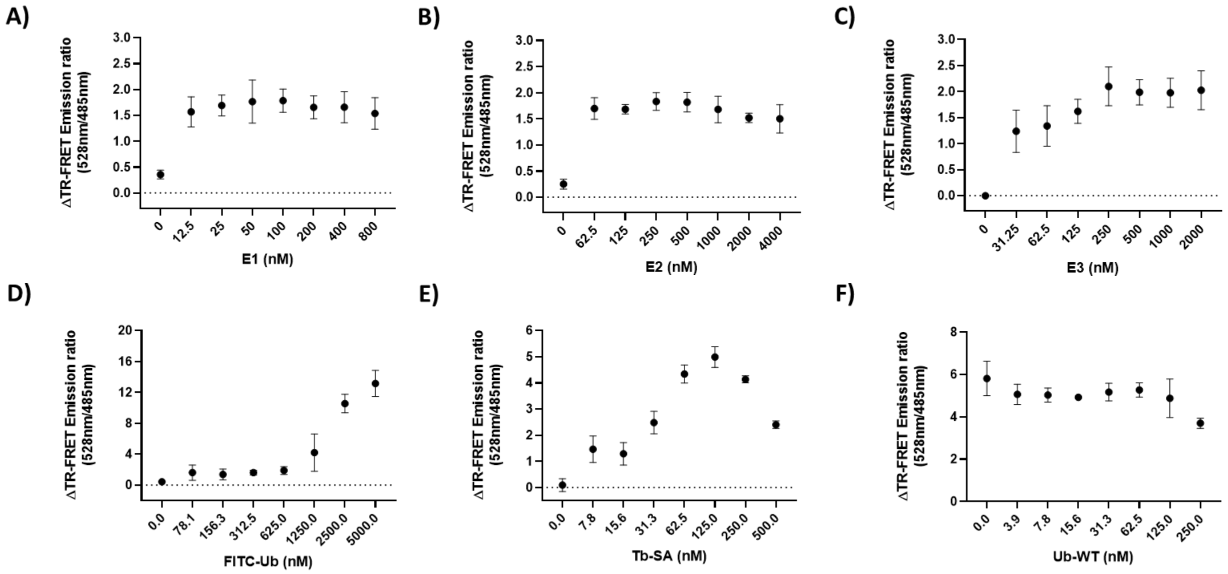

Figure S4. Determination of the suitability of the TR-FRET autoubiquitination for high-throughput screening for Nedd4. A) Plotting the ΔTR-FRET signal over reaction time indicates linear signal response up to the 60 min assay time that was used. **B)** Determination of the Z′-factor of 0.75 demonstrated suitability for high throughput screening.

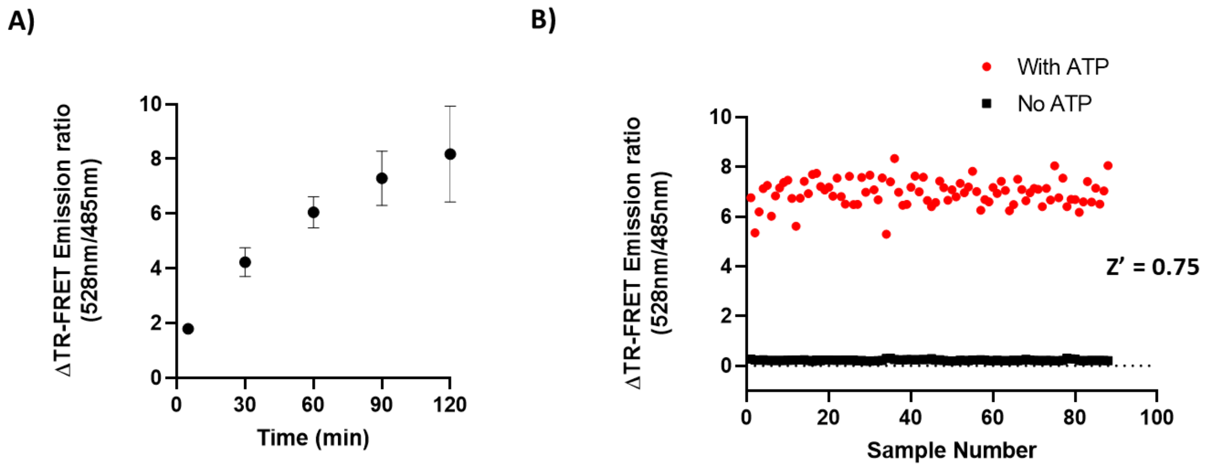

Figure S5. Testing TR-FRET autoubiquitination assay to determine Nedd4 activity in the presence of two previously reported Nedd4 inhibitors, NAB2 (7) and heclin (8). Compound structures are presented for both compounds on related plots. Data were analyzed using 4-parameter non-linear regression and the relative IC_{50} ± standard deviation is shown on each plot. Percent activity was calculated as ratio of the background subtracted TR-FRET signal at any given concentration of a potential inhibitor and the background subtracted TR-FRET signal in the absence of compound.

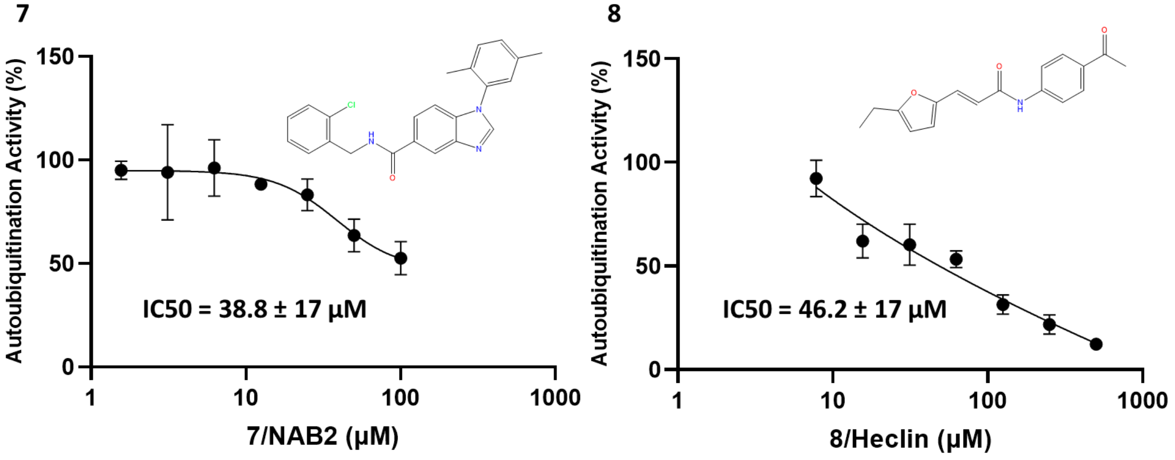

Figure S6. Sequence alignment of full length human Nedd4 and two related HECT domain containing E3 ubiquitin ligases. Cysteine sites found to be modified by either compound **25** or **81** are highlighted as follows: C182 in green, C627 in blue, and C867 in yellow. Only the residue corresponding to Nedd4 C867 is conserved in the sequences of all three family members. Cysteine sites not confirmed to be subject to **25** or **81** adduct formation in Nedd4 are highlighted in gray. Sequence accession information: Nedd4 (NCBI Reference Sequence: NP_006145.2), WWP1 (NCBI Reference Sequence: NP_008944.1), WWP2 (NCBI Reference Sequence: NP_001257383.1)

```
Nedd4      MATCAVEVFGLLEDEENSRIVRVRVIAGIGLAKKDILGASDPYVRVTLYDPMNGVLT-SV     59
WWP1       MATASPRSDTSNNHSGRLQ-LQVTVSSA-KLKRKKNWFGTAIYTEVV----VDGEI---T     51
WWP2       MASASSSRAGVALPFEKSQ-LTLKVVSA-KPKVHNRQPRINSYVEVA----VDGLPSETK     54
           **:.:           . : : : * :.     :.       *..*.    ::*

Nedd4      QTKTIKKSLNPKWNEEILFRVHPQQHRLLFEVFDENRLTRDDFLGQVDVPLYPLPTEN-P     118
WWP1       KTAKSSSSSNPKWDEQLTVNVTPQTT-LEFQVWSHRTLKADALLGKATIDLKQALLIHNR     110
WWP2       KTGKRIGSSELLWNEIIILNVTAQSH-LDLKVWSCHTLRN-ELLGTASVNLSNVLKNNGG     112
           :* .   * :  *:* : ..*  *   * ::*:. . *    :** . : *      :

Nedd4      RLERPYTFKDFVLHPRSHKSRVKGYLRL-------KMTYLPKTSGSED-----------D     160
WWP1       KLERVKEQLKLSLENKNG-IAQTGELTVVLDGLVIEQENITNCSSSPTIEIQENGDALHE     169
WWP2       KMENMQLTLNLQTENKGS-VVSGGELTIFLDGPTVDLGNVPNGSALT------------D     159
           ::*.     .:  . :.      * * :       .   : : *.              :

Nedd4      NAEQAEELEPGWVVLDQPDAACHLQQQQEPSPLPPGWEERQ----DILGRTYYVNHESRR     216
WWP1       NGEPSARTTARLAVEGT----NGIDNHVPTSTLVQN-----------SCCSYVVNGDNTP     214
WWP2       GSQLPSRDSSGTAVAPE-------NRHQPPSTNCFGGRSRTHRHSGASARTTPATGEQSP     212
           ..:   .     .*          :.:   *    .              :  .. :.

Nedd4      T------Q--WKRPTPQDNLTDAENGNIQLQAQRAFTTRRQISEETESVDNRESSENWEI     268
WWP1       SSPSQVAARPKNTPAPKPLASEPADDTVNGESS-SFAPTD-----NASV------TGTPV     262
WWP2       GARSRH-----RQPVKNSGHSGLANGTVNDEPT-TATDPE-----EPSV------V----     251
                      . *. :   :   :..:: :   : :          **

Nedd4      IREDEAT--MYSNQAFPSPPPSSNL--------DVPTHLAEELNARLTIFGNS-------     311
WWP1       VSEENALSPNCTSTTVEDPPVQEIL-TSSENNECIPSTSAELESEARSILEPDTSNSRSS     321
WWP2       --------------GVTSPPAAPLSVTPNPNTTSLPAPATPAEGE-----EPSTS-----     287
                          . .**              :*:  :   .        .

Nedd4      ------AVSQP----------ASSSN-------HSSRRGSLQAYTFEEQPTLPVLLPTSS     348
WWP1       SAFEAAKSRQPDGCMDPVRQQSGNANTETLPSGWEQRKDPHGRTYYVDHNTRTTTWERPQ     381
WWP2       ---------------GTQQLPAAAQAPDALPAGWEQRELPNGRVYYVDHNTKTTTWER--     330
                                :.           ..*.       : :: *  .

Nedd4      GLPPGWEEKQDERGRSYYVDHNSRTTTWTKPTVQATVETSQLTSSQSSAGPQSQA-S-TS     406
WWP1       PLPPGWERRVDDRRRVYYVDHNTRTTTWQRPTMESVRNFEQWQSQRNQLQGAMQQFNQRY     441
WWP2       PLPPGWEKRTDPRGRFYYVDHNTRTTTWQRPTAEYVRNYEQWQSQRNQLQGAMQHFSQRF     390
            ******.: * * * ******:***** :** : . : .*  *.:..     *  .

Nedd4      DSGQQVTQPSEIEQGFLPKGWEVRHAPNGRPFFIDHNTKTTTWEDPRLKIPAHLRGKTSL     466
WWP1       LYSASMLAAENDPYGPLPPGWEKRVDSTDRVYFVNHNTKTTQWEDPRTQG----------     491
WWP2       LYQSSSASTDHDPLGPLPPGWEKRQD-NGRVYYVNHNTRTTQWEDPRTQG----------     439
               .    ..   * ** *** *   ..* ::::***:** ***** :

Nedd4      DTSNDLGPLPPGWEERTHTDGRIFYINHNIKRTQWEDPRLE--NVAITGPAVPYSRDYKR     524
WWP1       --LQNEEPLPEGWEIRYTREGVRYFVDHNTRTTTFKDPRNGKSSVTKGGPQIAYERGFRW     549
WWP2       --MIQEPALPPGWEMKYTSEGVRYFVDHNTRTTTFKDPRPGFESGTKQGSPGAYDRSFRW     497
               :   ** *** :   :*  ::::** : * ::***    . :  *    *.*.::
```

```
Nedd4      KYEFFRRKLKKQNDIPNKFEMKLRRATVLEDSYRRIMGVKRADFLKARLWIEFDGEKGLD      584
WWP1       KLAHFRY-LCQSNALPSHVKINVSRQTLFEDSFQQIMALKPYD-LRRRLYVIFRGEEGLD      607
WWP2       KYHQFRF-LCHSNALPSHVKISVSRQTLFEDSFQQIMNMKPYD-LRRRLYIIMRGEEGLD      555
           *   **  * :.* :*.:.::.: * *::***:::** :*  * *: **:: : **:***

Nedd4      YGGVAREWFFLISKEMFNPYYGLFEYSATDNYTLQINPNSGLCNEDHLSYFKFIGRVAGM      644
WWP1       YGGLAREWFFLLSHEVLNPMYCLFEYAGKNNYCLQINPASTI-NPDHLSYFCFIGRFIAM      666
WWP2       YGGIAREWFFLLSHEVLNPMYCLFEYAGKNNYCLQINPASSI-NPDHLTYFRFIGRFIAM      614
           ***:*******:*:*::** * ****:..:** ***** * : * ***:** ****. .*

Nedd4      AVYHGKLLDGFFIRPFYKMMLHKPITLHDMESVDSEYYNSLRWILENDPTE--LDLRFII      702
WWP1       ALFHGKFIDTGFSLPFYKRMLSKKLTIKDLESIDTEFYNSLIWIRDNNIEECGLEMYFSV      726
WWP2       ALYHGKFIDTGFTLPFYKRMLNKRPTLKDLESIDPEFYNSIVWIKENNLEECGLELYFIQ      674
           *::***::*  *  **** ** *  *::*:**:* *:***: ** :*:  *  *:: *

Nedd4      DEELFGQTHQHELKNGGSEIVVTNKNKKEYIYLVIQWRFVNRIQKQMAAFKEGFFELIPQ      762
WWP1       DMEILGKVTSHDLKLGGSNILVTEENKDEYIGLMTEWRFSRGVQEQTKAFLDGFNEVVPL      786
WWP2       DMEILGKVTTHELKEGGESIRVTEENKEEYIMLLTDWRFTRGVEEQTKAFLDGFNEVAPL      734
           * *::*:.  *:** **..* **::**.*** *: :*** . :::*  ** :** *: *

Nedd4      DLIKIFDENELELLMCGLGDVDVNDWREHTKYKNGYSANHQVIQWFWKAVLMMDSEKRIR      822
WWP1       QWLQYFDEKELEVMLCGMQEVDLADWQRNTVYR-HYTRNSKQIIWFWQFVKETDNEVRMR      845
WWP2       EWLRYFDEKELELMLCGMQEIDMSDWQKSTIYR-HYTKNSKQIQWFWQVVKEMDNEKRIR      793
           : :: ***:***:::**: ::*: **:. * *:  *: * : * ***: *   *.* *:*

Nedd4      LLQFVTGTSRVPMNGFAELYGSNGPQSFTVEQWGTPEKLPRAHTCFNRLDLPPYESFEEL      882
WWP1       LLQFVTGTCRLPLGGFAELMGSNGPQKFCIEKVGKDTWLPRSHTCFNRLDLPPYKSYEQL      905
WWP2       LLQFVTGTCRLPVGGFAELIGSNGPQKFCIDKVGKETWLPRSHTCFNRLDLPPYKSYEQL      853
           ********.*:*:.***** ******.* ::: *.   ***:************:*:*:*

Nedd4      WDKLQMAIENTQGFDGVD     900
WWP1       KEKLLFAIEETEGFGQE-     922
WWP2       REKLLYAIEETEGFGQE-     870
            :**  ***:*:**.
```

Protein Purification Protocols and Sequence Information

Expression and Purification of Nedd4 Constructs

Constructs and Expression

DNA fragments encoding Nedd4 (full length wild type, amino acids153-900, 153-900 with deletion between 225-244, full length C182A, C627A, C867A, and C627A + C867A mutants) were amplified by PCR and sub-cloned into the pNicBio2 vector downstream of the poly-histidine coding region and upstream of an Avi-tag. Following transformation into *E. Coli BL21 (D3)-BirA* the target protein was over-expressed at 37 °C by inoculating Terrific Broth with overnight culture, supplemented with 50 µg/ml Kanamycin and 34 µg/ml chloramphenicol. When the OD_{600} of the culture reached 0.8-1.6, the temperature was lowered to 18 °C, the culture was induced with 1.0 mM IPTG (isopropyl-1-thio-D-galactopyranoside) and D-Biotin at 10 µg/mL and incubated overnight before being harvested (12,000 × g for 10 min at 10 °C) using a Beckman Coulter centrifuge (Avanti J20XP1).

Harvest and cell lysis

Harvested cells were resuspended in 50 mM Tris-HCl, pH 7.5 containing 500 mM NaCl, 5 mM imidazole and 5% glycerol, 1 × protease inhibitor cocktail (100 × protease inhibitor stock in 70% ethanol (0.25 mg/ml Aprotinin, 0.25 mg/ml Leupeptin, 0.25 mg/ml Pepstatin A and 0.25 mg/ml E-64) or Roche complete EDTA-free protease inhibitor cocktail tablet and 1 mM TCEP, CHAPS (final concentration of 0.5%) and 5 µl/L Benzonase nuclease (in house) followed by homogenization and sonication at frequency of 8.0 (10" on/7" off) for 10 min (Sonicator 3000, Misoni). The crude extract was clarified by high-speed centrifugation (45 min at 36,000 × g at 10 °C) by Beckman Coulter Centrifuge (Avanti J20XP1).

Protein Purification

The clarified lysate was then loaded onto an open column containing Ni^{2+}-NTA (Qiagen) pre-equilibrated with 50 mM Tris-HCl, pH 7.5, 500 mM NaCl, 5 mM imidazole and 5% glycerol. The column was washed with 1 full column of 1 mM D-biotin in PBS before being washed with 50 mM Tris-HCl, pH 7.5 containing 500 mM NaCl, 30 mM imidazole and 5% glycerol. The protein was then eluted by 50 mM Tris-HCl, pH 7.5, 500 mM NaCl, 5% glycerol, and 250 mM imidazole. The eluent was further purified by gel filtration on a Superdex200 26/600 using an ÄKTA PURE (Cytiva) pre-equilibrated with 50 mM Tris pH 7.5, 150 mM NaCl, 5% glycerol, and 1 mM TCEP. Finally, the purity of the fractions was confirmed on an SDS-PAGE gel and the pure fractions were pooled, concentrated and flash frozen.

The full-length wild type Nedd4, the truncated 153-900 Nedd4, and the 153-900 truncated Nedd4 with a deletion between 225-244, were additionally subjected to ion-exchange chromatography following size-exclusion chromatography (Resource Q, Cytiva) to improve the purity of the

protein. The purity of the fractions was confirmed through SDS-PAGE and the pure fractions were pooled and flash frozen.

Purification of Ube1

Constructs and Expression

UBE1 DNA fragment encoding 1-1058 amplified by PCR and sub-cloned into pFBOH-LIC downstream of His-tag. The resulting plasmid was transformed into DH10Bac™ Competent *E. coli* (Invitrogen) and a recombinant viral DNA bacmid was purified and followed by a recombinant baculovirus generation in *Sf*9 insect cells. *Sf*9 cells grown in HyQ® SFX insect serum-free medium (ThermoScientific) were infected with 10 ml of P3 viral stock per 0.8L of suspension cell culture incubated at 27 °C using a platform shaker set at 100 RPM. The cells were collected after 72 hours of post infection time, when viability dropped to 70-80%.

Harvest and cell lysis

Harvested cells were re-suspended in 20 mM Tris-HCl buffer, pH 7.5, containing 500 mM NaCl, 5 mM imidazole and 5% glycerol, 1 × protease inhibitor cocktail (100 × protease inhibitor stock in 70% ethanol (0.25 mg/ml Aprotinin, 0.25 mg/ml Leupeptin, 0.25 mg/ml Pepstatin A and 0.25 mg/ml E-64) or Roche complete EDTA-free protease inhibitor cocktail tablet. The cells were lysed chemically by rotating 30 min with NP40 (final concentration of 0.6%), 120 μl/L of Benzonase nuclease (in house) followed by sonication at frequency of 7.5 (10” on/10” off) for 2 min (Sonicator 3000, Misoni). The crude extract was clarified by high-speed centrifugation (60 min at 36,000 × g at 4 °C) by Beckman Coulter centrifuge.

Protein Purification

The clarified lysate was then loaded onto an open column containing pre-equilibrated Ni^{2+}-NTA (Qiagen). The column was washed and eluted by running 20 mM Tris-HCl, pH 7.5, 500 mM NaCl, 5% glycerol, containing 20 mM and 250 mM imidazole, respectively. Thrombin (Sigma) was added while the protein was dialyzing against 20 mM Tris-HCl,pH 7.5, 500 mM NaCl and 2.5 mM $CaCl_2$. In the next step, to remove His-tag and His-tagged proteins, dialyzed protein solution was loaded onto Ni^{2+}-NTA resin. The flow through dialyzed against 50 mM Tris-HCl pH 7.5, 300 mM NaCl, 1 mM DTT. The purity was confirmed on SDS-PAGE gels then concentrated and flash frozen.

Expression of UbcH5a

Constructs and Expression

DNA fragment encoding UbcH5a residues 1-147 was amplified by PCR and sub-cloned into pET28-MHL vector, downstream of the poly-histidine coding region. Following transformation into *E. coli* BL21 (DE3) the target protein was over-expressed at 37 °C by inoculating Terrific Broth with overnight culture, both supplemented with 50 µg/ml Kanamycin and 35 µg/ml chloramphenicol. When the OD_{600} of the culture reached 0.8- 1.5, the temperature was lowered to 18 °C, the culture was induced with 0.5 mM IPTG (isopropyl-1-thio-D-galactopyranoside)

and incubated overnight before being harvested (12,000 × g for 10 min at 4 °C) using a Beckman Coulter centrifuge.

Harvest and cell lysis

Harvested cells were re-suspended in 50 mM Tris-HCl buffer, pH 7.5, containing 500 mM NaCl, 5 mM imidazole and 5% glycerol, 1 × protease inhibitor cocktail (100 × protease inhibitor stock in 70% ethanol (0.25 mg/ml Aprotinin, 0.25 mg/ml Leupeptin, 0.25 mg/ml Pepstatin A and 0.25 mg/ml E-64) or Roche complete EDTA-free protease inhibitor cocktail table and 5 mM 2-Mercaptoethanol. The cells were lysed chemically by rotating 30 min with CHAPS (final concentration of 0.5%) and 5 µl/L Benzonase Nuclease (in house) followed by sonication at frequency of 8.0 (10" on/10" off) for 4 min (Sonicator 3000, Misoni). The crude extract was clarified by high-speed centrifugation (60 min at 36,000 × g at 4 °C) by Beckman Coulter centrifuge.

Protein Purification

The clarified lysate was then loaded onto an open column containing pre-equilibrated Ni^{2+}-NTA (Qiagen). The column was washed and eluted by running 50 mM Tris-HCl, pH 7.5, 500 mM NaCl, 5% glycerol, containing 30 mM and 250 mM imidazole, respectively. The UbcH5a protein was further purified by gel filtration on a Superdex200 26/600 using an ÄKTA PURE (GE Healthcare) pre-equilibrated with 50 mM Tris-HCl, pH 8.0, 150 mM NaCl 5% glycerol and 5 mM 2-Mercaptoethanol. The purity of the fractions was confirmed on SDS-PAGE gels and the pure fractions were pooled. The TEV (in-house) was added to the pure protein and incubated overnight. To remove His-tag and the remaining His-tagged protein, the mixture was loaded onto pre-equilibrated Ni^{2+}-NTA resin, and the untagged protein or the flow-through was concentrated, and flash frozen after confirming the correct size on the SDS PAGE and mass spectrometry.

Expression and Purification of UbcH7

Constructs and Expression

DNA fragment encoding UbcH7 residues 1-154 was amplified by PCR and sub-cloned into the pET28a-LIC vector downstream of the poly-histidine coding region. Following transformation into *E. coli* BL21 (DE3) the target protein was over-expressed at 37 °C by inoculating Terrific Broth with overnight culture, both supplemented with 50 µg/ml Kanamycin and 35 µg/ml chloramphenicol. When the OD600 of the culture reached 0.8- 1.5, the temperature was lowered to 18 °C, the culture was induced with 1 mM IPTG (isopropyl-1-thio-D-galactopyranoside) and incubated overnight before being harvested (12,000 × g for 10 min at 4 °C) using a Beckman Coulter centrifuge.

Harvest and cell lysis

Harvested cells were re-suspended in 20 mM Tris-HCl buffer, pH 7.5, containing 500 mM NaCl, 5 mM imidazole and 5% glycerol, 1 × protease inhibitor cocktail (100 × protease inhibitor stock in 70% ethanol (0.25 mg/ml Aprotinin, 0.25 mg/ml Leupeptin, 0.25 mg/ml Pepstatin A and 0.25 mg/ml E-64) or Roche complete EDTA-free protease inhibitor cocktail tablet. The cells were lysed

chemically by rotating 30 min with CHAPS (final concentration of 0.5%) and 5 µl/L Benzonase Nuclease (Sigma) followed by sonication at frequency of 8 (10” on/10” off) for 4 min (Sonicator 3000, Misoni). The crude extract was clarified by high-speed centrifugation (60 min at 36,000 × g at 4 °C) by Beckman Coulter centrifuge.

Protein Purification

The clarified lysate was then loaded onto an open column containing pre-equilibrated Ni^{2+}-NTA (Qiagen). The column was washed and eluted by running 20 mM Tris-HCl, pH 7.5, 500 mM NaCl, 5% glycerol, containing 20 mM and 250 mM imidazole, respectively. The thrombin enzyme (Sigma) was then added while the protein was dialyzing against 20 mM Tris-HCl (pH 7.5), 500 mM NaCl and 2.5 mM $CaCl_2$. In the next step, to remove His-tag and the remaining His-tagged proteins, dialyzed protein solution was loaded onto Ni^{2+}-NTA resin. The flow through was dialyzed against 50 mM Tris pH 7.5, 300 mM NaCl, 1 mM DTT. The purity was confirmed on SDS-PAGE gels, then concentrated and flash frozen.

Expression and Purification of WWP1

Constructs and Expression

DNA fragment encoding human WWP1 (residues 349- 922) was amplified by PCR and sub-cloned into a pET28-MHL vector, downstream of the poly-histidine coding region. Following transformation into *E. Coli* BL21 (DE3) the cells were amplified at 37°C by inoculating Terrific Broth with overnight culture, both supplemented with 50 µg/ml Kanamycin and 35 µg/ml chloramphenicol. When the OD600 of the culture reached 0.8- 1.5, the temperature was lowered to 16 °C and the target protein was over-expressed by inducing cells with 0.5 mM IPTG (isopropyl-1-thio-D-galactopyranoside) and incubated overnight before being harvested (12,000 × g for 10 min at 4 °C) using a Beckman Coulter centrifuge.

Harvest and cell lysis

Harvested cells were re-suspended in 20 mM Tris-HCl, pH 7.5, 500 mM NaCl, 5 mM imidazole and 5% glycerol, 1 × protease inhibitor cocktail (100 × protease inhibitor stock in 70% ethanol (0.25 mg/ml Aprotinin, 0.25 mg/ml Leupeptin, 0.25 mg/ml Pepstatin A and 0.25 mg/ml E-64) or Pierce™ Protease Inhibitor Mini Tablets, EDTA-free. The cells were lysed chemically by rotating 30 min with 0.5% CHAPS, 1 mM DTT, and 15 µL Benzonase Nuclease (In-House) followed by sonication at frequency of 8.0 (5” on/7” off) for 5 min (Sonicator 3000, Misoni). The crude extract was clarified by high-speed centrifugation (60 min at 36,000 × g at 4 °C) by Beckman Coulter centrifuge.

Protein Purification

The clarified lysate was then loaded onto an open column containing pre-equilibrated Ni^{2+}-NTA (Qiagen). The column was first washed with binding buffer, then washed and eluted by running 20 mM Tris-HCl, pH 7.5, 500 mM NaCl, 5% glycerol, containing 20 mM and 250 mM imidazole, respectively. The eluted protein was then supplemented with 1 mM DTT and concentrated to be further purified by gel filtration on a HiLoad Superdex200 26/600 using an ÄKTA Pure (GE

Healthcare). The gel filtration column was pre-equilibrated with 20 mM Tris-HCl, pH 7.5, 200 mM NaCl, 1 mM DTT, 5% glycerol. The purity of the fractions was assessed on SDS-PAGE gels and pure fractions were pooled, concentrated and flash frozen.

<u>Expression and Purification of WWP2</u>

Constructs and Expression

DNA fragment encoding WWP2 (residues M 1-E 870, isoform 1) was amplified by PCR and sub-cloned into pFBD-BirA vector (a derivative of pFastBac Dual vector, Invitrogen) downstream of the Avi-Tag for in cell biotinylation and a C-terminal 6× His tag. The resulting plasmid was transformed into DH10Bac™ Competent E. Coli (Invitrogen) and a recombinant viral DNA bacmid was purified and followed by a recombinant baculovirus generation in *Sf9* insect cells. The *Sf9* cells were grown in HyQ® SFX insect serum-free medium (ThermoScientific) and infected with 10 ml of P3 viral stock per 0.8 L of suspension cell culture and incubated at 27 °C using a platform shaker set at 100 RPM. The cells were collected after 72 hours post infection time, when viability dropped to 70-80%.

Harvest and cell lysis

Harvested cells were re-suspended in 20 mM Tris-HCl,pH 7.5 containing 500 mM NaCl, 5 mM imidazole, 5% glycerol, 1 mM TCEP, 1 × protease inhibitor cocktail (100 × protease inhibitor stock in 70% ethanol containing 0.25 mg/ml Aprotinin, 0.25 mg/ml Leupeptin, 0.25 mg/ml Pepstatin A and 0.25 mg/ml E-64) and Pierce complete EDTA-free protease inhibitor cocktail tablet. The cells were lysed chemically by rotating for 30 min with NP40 (final concentration of 0.5%), 22.5 U/mL Benzonase nuclease (in house) and 1 mM TCEP followed by sonication at frequency of 7.5 (10” on/10” off) for 6 min (Sonicator 3000, Misoni). The crude extract was clarified by high-speed centrifugation (60 min at 28,000 × g at 4˚C) by Beckman Coulter centrifuge.

Protein Purification

The cleared lysate was loaded onto Ni^{2+}-NTA affinity resin column (Qiagen). The column was washed with 50 mM Tris-HCl,pH 8.0, 500 mM NaCl, 5% glycerol, 30 mM imidazole and 1 mM TCEP after washing with 1 mM biotin in PBS. The biotinylated WWP2 protein was then eluted in 50 mM Tris-HCl pH 8.0, 500 mM NaCl, 5% glycerol, 250 mM imidazole, 1 mM TCEP. To ensure that the WWP2 protein was highly biotinylated, the protein was further biotinylated through an *in vitro* BirA biotin ligase enzymatic reaction (Avidity, USA). The uncut protein was loaded onto a Superdex20026/600 column pre-equilibrated with 50 mM Tris-HCl pH 8.0, 150 mM NaCl, 5% glycerol, 1 mM TCEP. The purity of the fractions was confirmed on SDS-PAGE gel and the pure fractions were pooled and concentrated and flash frozen.

Protein Sequences

Nedd4 Full-Length

N-terminal tag: MHHHHHHHHHHDLGTENLYFQS

C-terminal tag: SKGGYGLNDIFEAQKIEWHE

Residues (from-to): 1-900

MHHHHHHHHHHDLGTENLYFQSMATCAVEVFGLLEDEENSRIVRVRVIAGIGLAKKDI
LGASDPYVRVTLYDPMNGVLTSVQTKTIKKSLNPKWNEEILFRVHPQQHRLLFEVFDEN
RLTRDDFLGQVDVPLYPLPTENPRLERPYTFKDFVLHPRSHKSRVKGYLRLKMTYLPKT
SGSEDDNAEQAEELEPGWVVLDQPDAACHLQQQQEPSPLPPGWEERQDILGRTYYVNH
ESRRTQWKRPTPQDNLTDAENGNIQLQAQRAFTTRRQISEETESVDNRESSENWEIIRED
EATMYSNQAFPSPPPSSNLDVPTHLAEELNARLTIFGNSAVSQPASSSNHSSRRGSLQAY
TFEEQPTLPVLLPTSSGLPPGWEEKQDERGRSYYVDHNSRTTTWTKPTVQATVETSQLT
SSQSSAGPQSQASTSDSGQQVTQPSEIEQGFLPKGWEVRHAPNGRPFFIDHNTKTTTWED
PRLKIPAHLRGKTSLDTSNDLGPLPPGWEERTHTDGRIFYINHNIKRTQWEDPRLENVAIT
GPAVPYSRDYKRKYEFFRRKLKKQNDIPNKFEMKLRRATVLEDSYRRIMGVKRADFLK
ARLWIEFDGEKGLDYGGVAREWFFLISKEMFNPYYGLFEYSATDNYTLQINPNSGLCNE
DHLSYFKFIGRVAGMAVYHGKLLDGFFIRPFYKMMLHKPITLHDMESVDSEYYNSLRWI
LENDPTELDLRFIIDEELFGQTHQHELKNGGSEIVVTNKNKKEYIYLVIQWRFVNRIQKQ
MAAFKEGFFELIPQDLIKIFDENELELLMCGLGDVDVNDWREHTKYKNGYSANHQVIQ
WFWKAVLMMDSEKRIRLLQFVTGTSRVPMNGFAELYGSNGPQSFTVEQWGTPEKLPR
AHTCFNRLDLPPYESFEELWDKLQMAIENTQGFDGVDSSKGGYGLNDIFEAQKIEWHE

Nedd4 153-900

N-terminal tag: MHHHHHHHHHHDLGTENLYFQS

C-terminal tag: SKGGYGLNDIFEAQKIEWHE

Residues (from-to): 153-900

MHHHHHHHHHHDLGTENLYFQSMKTSGSEDDNAEQAEELEPGWVVLDQPDAACHLQ
QQQEPSPLPPGWEERQDILGRTYYVNHESRRTQWKRPTPQDNLTDAENGNIQLQAQRA
FTTRRQISEETESVDNRESSENWEIIREDEATMYSNQAFPSPPPSSNLDVPTHLAEELNAR
LTIFGNSAVSQPASSSNHSSRRGSLQAYTFEEQPTLPVLLPTSSGLPPGWEEKQDERGRSY
YVDHNSRTTTWTKPTVQATVETSQLTSSQSSAGPQSQASTSDSGQQVTQPSEIEQGFLPK
GWEVRHAPNGRPFFIDHNTKTTTWEDPRLKIPAHLRGKTSLDTSNDLGPLPPGWEERTH
TDGRIFYINHNIKRTQWEDPRLENVAITGPAVPYSRDYKRKYEFFRRKLKKQNDIPNKFE
MKLRRATVLEDSYRRIMGVKRADFLKARLWIEFDGEKGLDYGGVAREWFFLISKEMFN
PYYGLFEYSATDNYTLQINPNSGLCNEDHLSYFKFIGRVAGMAVYHGKLLDGFFIRPFY
KMMLHKPITLHDMESVDSEYYNSLRWILENDPTELDLRFIIDEELFGQTHQHELKNGGSE
IVVTNKNKKEYIYLVIQWRFVNRIQKQMAAFKEGFFELIPQDLIKIFDENELELLMCGLG
DVDVNDWREHTKYKNGYSANHQVIQWFWKAVLMMDSEKRIRLLQFVTGTSRVPMNG
FAELYGSNGPQSFTVEQWGTPEKLPRAHTCFNRLDLPPYESFEELWDKLQMAIENTQGF
DGVDSSKGGYGLNDIFEAQKIEWHE

Nedd4 153-900 delta 225-244AQ

N-terminal tag: MHHHHHHHHHHDLGTENLYFQS

C-terminal tag: SKGGYGLNDIFEAQKIEWHE

Residues (from-to): 153-900

MHHHHHHHHHHDLGTENLYFQSMKTSGSEDDNAEQAEELEPGWVVLDQPDAACHLQ
QQQEPSPLPPGWEERQDILGRTYYVNHESRRTQWKRPTPTTRRQISEETESVDNRESSEN
WEIIREDEATMYSNQAFPSPPPSSNLDVPTHLAEELNARLTIFGNSAVSQPASSSNHSSRR
GSLQAYTFEEQPTLPVLLPTSSGLPPGWEEKQDERGRSYYVDHNSRTTTWTKPTVQATV
ETSQLTSSQSSAGPQSQASTSDSGQQVTQPSEIEQGFLPKGWEVRHAPNGRPFFIDHNTK
TTTWEDPRLKIPAHLRGKTSLDTSNDLGPLPPGWEERTHTDGRIFYINHNIKRTQWEDPR
LENVAITGPAVPYSRDYKRKYEFFRRKLKKQNDIPNKFEMKLRRATVLEDSYRRIMGVK
RADFLKARLWIEFDGEKGLDYGGVAREWFFLISKEMFNPYYGLFEYSATDNYTLQINPN
SGLCNEDHLSYFKFIGRVAGMAVYHGKLLDGFFIRPFYKMMLHKPITLHDMESVDSEY
YNSLRWILENDPTELDLRFIIDEELFGQTHQHELKNGGSEIVVTNKNKKEYIYLVIQWRF
VNRIQKQMAAFKEGFFELIPQDLIKIFDENELELLMCGLGDVDVNDWREHTKYKNGYSA
NHQVIQWFWKAVLMMDSEKRIRLLQFVTGTSRVPMNGFAELYGSNGPQSFTVEQWGT
PEKLPRAHTCFNRLDLPPYESFEELWDKLQMAIENTQGFDGVDSSKGGYGLNDIFEAQK
IEWHE

Nedd4 C182A

N-terminal tag: MHHHHHHHHHHDLGTENLYFQS

C-terminal tag: SKGGYGLNDIFEAQKIEWHE

Residues (from-to): 1-900

<u>MHHHHHHHHHHDLGTENLYFQS</u>MATCAVEVFGLLEDEENSRIVRVRVIAGIGLAKKDILGASDP
YVRVTLYDPMNGVLTSVQTKTIKKSLNPKWNEEILFRVHPQQHRLLFEVFDENRLTRDDFLGQV
DVPLYPLPTENPRLERPYTFKDFVLHPRSHKSRVKGYLRLKMTYLPKTSGSEDDNAEQAEELEPG
WVVLDQPDAAaHLQQQQEPSPLPPGWEERQDILGRTYYVNHESRRTQWKRPTPQDNLTDAENG
NIQLQAQRAFTTRRQISEETESVDNRESSENWEIIREDEATMYSNQAFPSPPPSSNLDVPTHLAEEL
NARLTIFGNSAVSQPASSSNHSSRRGSLQAYTFEEQPTLPVLLPTSSGLPPGWEEKQDERGRSYYV
DHNSRTTTWTKPTVQATVETSQLTSSQSSAGPQSQASTSDSGQQVTQPSEIEQGFLPKGWEVRHA
PNGRPFFIDHNTKTTTWEDPRLKIPAHLRGKTSLDTSNDLGPLPPGWEERTHTDGRIFYINHNIKR
TQWEDPRLENVAITGPAVPYSRDYKRKYEFFRRKLKKQNDIPNKFEMKLRRATVLEDSYRRIMG
VKRADFLKARLWIEFDGEKGLDYGGVAREWFFLISKEMFNPYYGLFEYSATDNYTLQINPNSGL
CNEDHLSYFKFIGRVAGMAVYHGKLLDGFFIRPFYKMMLHKPITLHDMESVDSEYYNSLRWILE
NDPTELDLRFIIDEELFGQTHQHELKNGGSEIVVTNKNKKEYIYLVIQWRFVNRIQKQMAAFKEG
FFELIPQDLIKIFDENELELLMCGLGDVDVNDWREHTKYKNGYSANHQVIQWFWKAVLMMDSE
KRIRLLQFVTGTSRVPMNGFAELYGSNGPQSFTVEQWGTPEKLPRAHTCFNRLDLPPYESFEELW
DKLQMAIENTQGFDGVD<u>SSKGGYGLNDIFEAQKIEWHE</u>

Nedd4 C627A

N-terminal tag: MHHHHHHHHHHDLGTENLYFQS

C-terminal tag: SKGGYGLNDIFEAQKIEWHE

Residues (from-to): 1-900

MHHHHHHHHHHDLGTENLYFQSMATCAVEVFGLLEDEENSRIVRVRVIAGIGLAKKDILGASDP
YYVRVTLYDPMNGVLTSVQTKTIKKSLNPKWNEEILFRVHPQQHRLLFEVFDENRLTRDDFLGQV
DVPLYPLPTENPRLERPYTFKDFVLHPRSHKSRVKGYLRLKMTYLPKTSGSEDDNAEQAEELEPG
WVVLDQPDAACHLQQQQEPSPLPPGWEERQDILGRTYYVNHESRRTQWKRPTPQDNLTDAENG
NIQLQAQRAFTTRRQISEETESVDNRESSENWEIIREDEATMYSNQAFPSPPPSSNLDVPTHLAEEL
NARLTIFGNSAVSQPASSSNHSSRRGSLQAYTFEEQPTLPVLLPTSSGLPPGWEEKQDERGRSYYV
DHNSRTTTWTKPTVQATVETSQLTSSQSSAGPQSQASTSDSGQQVTQPSEIEQGFLPKGWEVRHA
PNGRPFFIDHNTKTTTWEDPRLKIPAHLRGKTSLDTSNDLGPLPPGWEERTHTDGRIFYINHNIKR
TQWEDPRLENVAITGPAVPYSRDYKRKYEFFRRKLKKQNDIPNKFEMKLRRATVLEDSYRRIMG
VKRADFLKARLWIEFDGEKGLDYGGVAREWFFLISKEMFNPYYGLFEYSATDNYTLQINPNSGL
aNEDHLSYFKFIGRVAGMAVYHGKLLDGFFIRPFYKMMLHKPITLHDMESVDSEYYNSLRWILE
NDPTELDLRFIIDEELFGQTHQHELKNGGSEIVVTNKNKKEYIYLVIQWRFVNRIQKQMAAFKEG
FFELIPQDLIKIFDENELELLMCGLGDVDVNDWREHTKYKNGYSANHQVIQWFWKAVLMMDSE
KRIRLLQFVTGTSRVPMNGFAELYGSNGPQSFTVEQWGTPEKLPRAHTCFNRLDLPPYESFEELW
DKLQMAIENTQGFDGVDSSKGGYGLNDIFEAQKIEWHE

Nedd4 C867A

N-terminal tag: MHHHHHHHHHHDLGTENLYFQS

C-terminal tag: SKGGYGLNDIFEAQKIEWHE

Residues (from-to): 1-900

MHHHHHHHHHHDLGTENLYFQSMATCAVEVFGLLEDEENSRIVRVRVIAGIGLAKKDILGASDP
YVRVTLYDPMNGVLTSVQTKTIKKSLNPKWNEEILFRVHPQQHRLLFEVFDENRLTRDDFLGQV
DVPLYPLPTENPRLERPYTFKDFVLHPRSHKSRVKGYLRLKMTYLPKTSGSEDDNAEQAEELEPG
WVVLDQPDAACHLQQQQEPSPLPPGWEERQDILGRTYYVNHESRRTQWKRPTPQDNLTDAENG
NIQLQAQRAFTTRRQISEETESVDNRESSENWEIIREDEATMYSNQAFPSPPPSSNLDVPTHLAEEL
NARLTIFGNSAVSQPASSSNHSSRRGSLQAYTFEEQPTLPVLLPTSSGLPPGWEEKQDERGRSYYV
DHNSRTTTWTKPTVQATVETSQLTSSQSSAGPQSQASTSDSGQQVTQPSEIEQGFLPKGWEVRHA
PNGRPFFIDHNTKTTTWEDPRLKIPAHLRGKTSLDTSNDLGPLPPGWEERTHTDGRIFYINHNIKR
TQWEDPRLENVAITGPAVPYSRDYKRKYEFFRRKLKKQNDIPNKFEMKLRRATVLEDSYRRIMG
VKRADFLKARLWIEFDGEKGLDYGGVAREWFFLISKEMFNPYYGLFEYSATDNYTLQINPNSGL
CNEDHLSYFKFIGRVAGMAVYHGKLLDGFFIRPFYKMMLHKPITLHDMESVDSEYYNSLRWILE
NDPTELDLRFIIDEELFGQTHQHELKNGGSEIVVTNKNKKEYIYLVIQWRFVNRIQKQMAAFKEG
FFELIPQDLIKIFDENELELLMCGLGDVDVNDWREHTKYKNGYSANHQVIQWFWKAVLMMDSE
KRIRLLQFVTGTSRVPMNGFAELYGSNGPQSFTVEQWGTPEKLPRAHTaFNRLDLPPYESFEELW
DKLQMAIENTQGFDGVDSSKGGYGLNDIFEAQKIEWHE

Nedd4 C627A + C867A

N-terminal tag: MHHHHHHHHHHDLGTENLYFQS

C-terminal tag: SKGGYGLNDIFEAQKIEWHE

Residues (from-to): 1-900

MHHHHHHHHHHDLGTENLYFQSMATCAVEVFGLLEDEENSRIVRVRVIAGIGLAKKDILGASDP
YVRVTLYDPMNGVLTSVQTKTIKKSLNPKWNEEILFRVHPQQHRLLFEVFDENRLTRDDFLGQV
DVPLYPLPTENPRLERPYTFKDFVLHPRSHKSRVKGYLRLKMTYLPKTSGSEDDNAEQAEELEPG
WVVLDQPDAACHLQQQQEPSPLPPGWEERQDILGRTYYVNHESRRTQWKRPTPQDNLTDAENG
NIQLQAQRAFTTRRQISEETESVDNRESSENWEIIREDEATMYSNQAFPSPPPSSNLDVPTHLAEEL
NARLTIFGNSAVSQPASSSNHSSRRGSLQAYTFEEQPTLPVLLPTSSGLPPGWEEKQDERGRSYYV
DHNSRTTTWTKPTVQATVETSQLTSSQSSAGPQSQASTSDSGQQVTQPSEIEQGFLPKGWEVRHA
PNGRPFFIDHNTKTTTWEDPRLKIPAHLRGKTSLDTSNDLGPLPPGWEERTHTDGRIFYINHNIKR
TQWEDPRLENVAITGPAVPYSRDYKRKYEFFRRKLKKQNDIPNKFEMKLRRATVLEDSYRRIMG
VKRADFLKARLWIEFDGEKGLDYGGVAREWFFLISKEMFNPYYGLFEYSATDNYTLQINPNSGL
aNEDHLSYFKFIGRVAGMAVYHGKLLDGFFIRPFYKMMLHKPITLHDMESVDSEYYNSLRWILE
NDPTELDLRFIIDEELFGQTHQHELKNGGSEIVVTNKNKKEYIYLVIQWRFVNRIQKQMAAFKEG
FFELIPQDLIKIFDENELELLMCGLGDVDVNDWREHTKYKNGYSANHQVIQWFWKAVLMMDSE
KRIRLLQFVTGTSRVPMNGFAELYGSNGPQSFTVEQWGTPEKLPRAHTaFNRLDLPPYESFEELW
DKLQMAIENTQGFDGVDSSKGGYGLNDIFEAQKIEWHE

Uba1 (cut)

N-terminal tag: MGSSHHHHHHSSGLVPRGS

Residues (from-to): 1-1058

GSMGSSHHHHHHSSGLVPRGSMSSSPLSKKRRVSGPDPKPGSNCSPAQSVLSEVPSVPTN
GMAKNGSEADIDEGLYSRQLYVLGHEAMKRLQTSSVLVSGLRGLGVEIAKNIILGGVKA
VTLHDQGTAQWADLSSQFYLREEDIGKNRAEVSQPRLAELNSYVPVTAYTGPLVEDFLS
GFQVVVLTNTPLEDQLRVGEFCHNRGIKLVVADTRGLFGQLFCDFGEEMILTDSNGEQP
LSAMVSMVTKDNPGVVTCLDEARHGFESGDFVSFSEVQGMVELNGNQPMEIKVLGPYT
FSICDTSNFSDYIRGGIVSQVKVPKKISFKSLVASLAEPDFVVTDFAKFSRPAQLHIGFQAL
HQFCAQHGRPPRPRNEEDAAELVALAQAVNARALPAVQQNNLDEDLIRKLAYVAAGD
LAPINAFIGGLAAQEVMKACSGKFMPIMQWLYFDALECLPEDKEVLTEDKCLQRQNRY
DGQVAVFGSDLQEKLGKQKYFLVGAGAIGCELLKNFAMIGLGCGEGGEIIVTDMDTIEK
SNLNRQFLFRPWDVTKLKSDTAAAAVRQMNPHIRVTSHQNRVGPDTERIYDDDFFQNL
DGVANALDNVDARMYMDRRCVYYRKPLLESGTLGTKGNVQVVIPFLTESYSSSQDPPE
KSIPICTLKNFPNAIEHTLQWARDEFEGLFKQPAENVNQYLTDPKFVERTLRLAGTQPLE
VLEAVQRSLVLQRPQTWADCVTWACHHWHTQYSNNIRQLLHNFPPDQLTSSGAPFWS
GPKRCPHPLTFDVNNPLHLDYVMAAANLFAQTYGLTGSQDRAAVATFLQSVQVPEFTP
KSGVKIHVSDQELQSANASVDDSRLEELKATLPSPDKLPGFKMYPIDFEKDDDSNFHMD
FIVAASNLRAENYDIPSADRHKSKLIAGKIIPAIATTTAAVVGLVCLELYKVVQGHRQLD
SYKNGFLNLALPFFGFSEPLAAPRHQYYNQEWTLWDRFEVQGLQPNGEEMTLKQFLDY
FKTEHKLEITMLSQGVSMLYSFFMPAAKLKERLDQPMTEIVSRVSKRKLGRHVRALVLE
LCCNDESGEDVEVPYVRYTIR

Ubch5a (cut)

N-terminal tag: MHHHHHHSSGRENLYFQG

Residues (from-to): 1-147

GMALKRIQKELSDLQRDPPAHCSAGPVGDDLFHWQATIMGPPDSAYQGGVFFLTVHFPTDYPFK
PPKIAFTTKIYHPNINSNGSICLDILRSQWSPALTVSKVLLSICSLLCDPNPDDPLVPDIAQIYKSDK
EKYNRHAREWTQKYAM

UbcH7 (cut)

N-terminal tag: MGSSHHHHHHSSGLVPRGS

Residues (from-to): 1-154

GSMAASRRLMKELEEIRKCGMKNFRNIQVDEANLLTWQGLIVPDNPPYDKGAFRIEINF
PAEYPFKPPKITFKTKIYHPNIDEKGQVCLPVISAENWKPATKTDQVIQSLIALVNDPQPE
HPLRADLAEEYSKDRKKFCKNAEEFTKKYGEKRPVD

WWP1 349-922

N-terminal tag: MHHHHHHSSGRENLYFQG

Residues (from-to): 349- 922

MHHHHHHSSGRENLYFQGETLPSGWEQRKDPHGRTYYVDHNTRTTTWERPQPLPPGW
ERRVDDRRRVYYVDHNTRTTTWQRPTMESVRNFEQWQSQRNQLQGAMQQFNQRYLYS
ASMLAAENDPYGPLPPGWEKRVDSTDRVYFVNHNTKTTQWEDPRTQGLQNEEPLPEG
WEIRYTREGVRYFVDHNTRTTTFKDPRNGKSSVTKGGPQIAYERGFRWKLAHFRYLCQS
NALPSHVKINVSRQTLFEDSFQQIMALKPYDLRRRLYVIFRGEEGLDYGGLAREWFFLLS
HEVLNPMYCLFEYAGKNNYCLQINPASTINPDHLSYFCFIGRFIAMALFHGKFIDTGFSLP
FYKRMLSKKLTIKDLESIDTEFYNSLIWIRDNNIEECGLEMYFSVDMEILGKVTSHDLKLG
GSNILVTEENKDEYIGLMTEWRFSRGVQEQTKAFLDGFNEVVPLQWLQYFDEKELEVM
LCGMQEVDLADWQRNTVYRHYTRNSKQIIWFWQFVKETDNEVRMRLLQFVTGTCRLP
LGGFAELMGSNGPQKFCIEKVGKDTWLPRSHTCFNRLDLPPYKSYEQLKEKLLFAIEETE
GFGQE

WWP2

N-terminal tag: MSGLNDIFEAQKIEWHEGSAGGSG

C-terminal tag: GGSGHHHHHH

Residues (from-to): 1-870

MSGLNDIFEAQKIEWHEGSAGGSGMASASSSRAGVALPFEKSQLTLKVVSAKPKVHNR
QPRINSYVEVAVDGLPSETKKTGKRIGSSELLWNEIIILNVTAQSHLDLKVWSCHTLRNE
LLGTASVNLSNVLKNNGGKMENMQLTLNLQTENKGSVVSGGELTIFLDGPTVDLGNVP
NGSALTDGSQLPSRDSSGTAVAPENRHQPPSTNCFGGRSRTHRHSGASARTTPATGEQSP
GARSRHRQPVKNSGHSGLANGTVNDEPTTATDPEEPSVVGVTSPPAAPLSVTPNPNTTSL
PAPATPAEGEEPSTSGTQQLPAAAQAPDALPAGWEQRELPNGRVYYVDHNTKTTTWER
PLPPGWEKRTDPRGRFYYVDHNTRTTTWQRPTAEYVRNYEQWQSQRNQLQGAMQHFS
QRFLYQSSSASTDHDPLGPLPPGWEKRQDNGRVYYVNHNTRTTQWEDPRTQGMIQEPA
LPPGWEMKYTSEGVRYFVDHNTRTTTFKDPRPGFESGTKQGSPGAYDRSFRWKYHQFR
FLCHSNALPSHVKISVSRQTLFEDSFQQIMNMKPYDLRRRLYIIMRGEEGLDYGGIAREW
FFLLSHEVLNPMYCLFEYAGKNNYCLQINPASSINPDHLTYFRFIGRFIAMALYHGKFIDT
GFTLPFYKRMLNKRPTLKDLESIDPEFYNSIVWIKENNLEECGLELYFIQDMEILGKVTTH
ELKEGGESIRVTEENKEEYIMLLTDWRFTRGVEEQTKAFLDGFNEVAPLEWLRYFDEKE
LELMLCGMQEIDMSDWQKSTIYRHYTKNSKQIQWFWQVVKEMDNEKRIRLLQFVTGT
CRLPVGGFAELIGSNGPQKFCIDKVGKETWLPRSHTCFNRLDLPPYKSYEQLREKLLYAI
EETEGFGQEGGSGHHHHHH
